# Genome-wide analysis of the maternal-to-zygotic transition in *Drosophila *primordial germ cells

**DOI:** 10.1186/gb-2012-13-2-r11

**Published:** 2012-02-20

**Authors:** Najeeb U Siddiqui, Xiao Li, Hua Luo, Angelo Karaiskakis, Huayun Hou, Thomas Kislinger, J Timothy Westwood, Quaid Morris, Howard D Lipshitz

**Affiliations:** 1 Department of Molecular Genetics, University of Toronto, 1 King's College Circle, Toronto, Ontario, Canada M5S 1A8; 2 Program in Developmental and Stem Cell Biology, Hospital for Sick Children Research Institute, Toronto Medical Discovery Tower, 101 College Street, Toronto, Ontario, Canada M5G 1L7; 3 School of Life Sciences, Peking University, No.5 Yiheyuan Road, Haidian District, Beijing, China 100871; 4 University Health Network and Department of Medical Biophysics, University of Toronto, Toronto Medical Discovery Tower, 101 College Street, Toronto, Ontario, Canada M5G 1L7; 5 Department of Cell and Systems Biology and Department of Biology, University of Toronto at Mississauga, 3359 Mississauga Road, Mississauga, Ontario, Canada L5L 1C6; 6 Banting and Best Department of Medical Research, Terrence Donnelly Centre for Cellular and Biomolecular Research, 160 College Street, Toronto, Ontario, Canada M5S 3E1

## Abstract

**Background:**

During the maternal-to-zygotic transition (MZT) vast changes in the embryonic transcriptome are produced by a combination of two processes: elimination of maternally provided mRNAs and synthesis of new transcripts from the zygotic genome. Previous genome-wide analyses of the MZT have been restricted to whole embryos. Here we report the first such analysis for primordial germ cells (PGCs), the progenitors of the germ-line stem cells.

**Results:**

We purified PGCs from *Drosophila *embryos, defined their proteome and transcriptome, and assessed the content, scale and dynamics of their MZT. Transcripts encoding proteins that implement particular types of biological functions group into nine distinct expression profiles, reflecting coordinate control at the transcriptional and posttranscriptional levels. mRNAs encoding germ-plasm components and cell-cell signaling molecules are rapidly degraded while new transcription produces mRNAs encoding the core transcriptional and protein synthetic machineries. The RNA-binding protein Smaug is essential for the PGC MZT, clearing transcripts encoding proteins that regulate stem cell behavior, transcriptional and posttranscriptional processes. Computational analyses suggest that Smaug and AU-rich element binding proteins function independently to control transcript elimination.

**Conclusions:**

The scale of the MZT is similar in the soma and PGCs. However, the timing and content of their MZTs differ, reflecting the distinct developmental imperatives of these cell types. The PGC MZT is delayed relative to that in the soma, likely because relief of PGC-specific transcriptional silencing is required for zygotic genome activation as well as for efficient maternal transcript clearance.

## Background

During early animal embryogenesis the maternal-to-zygotic transition (MZT) accomplishes two things: elimination of a subset of maternally encoded gene products and transcriptional activation of the zygote's genome. The scale of these two processes is very similar in all animals, with about a third of the maternal mRNAs being cleared from embryos and about a fifth of the zygote's genome being transcribed at high levels (reviewed in [1,2]). While the scale of these events is highly conserved, the choice of specific transcripts for either elimination or production differs extensively, probably because the vast differences in cellular and developmental processes in early embryos of different phyla impose distinct requirements on the composition of their transcriptome and proteome.

The regulatory mechanisms and roles of specific proteins and small RNAs in the MZT have been elucidated for several animal species. In *Drosophila*, for example, the sequence-specific RNA-binding protein (RBP) Smaug is translated in early embryos and is required for elimination of two-thirds of the destabilized maternal transcripts [3]. Smaug binds to stem-loop structures known as Smaug recognition elements (SREs) and functions to translationally repress and/or destabilize its direct targets, the latter via recruitment of the CCR4/POP2/NOT-deadenylase complex [4-9]. Smaug's role in post-transcriptional regulation is conserved from budding yeast [5] to humans [10,11].

In *Drosophila smaug *mutants, not only does maternal mRNA clearance not occur but zygotic genome activation fails [12]. This has led to the hypothesis that the latter effect is indirect and results from failure to downregulate maternally encoded transcriptional repressors which, in turn, is required for high-level zygotic genome activation. Among the genes that fail to be activated in *smaug *mutants are the *miR-309*-cluster microRNAs (*miR*s), which normally feed back to further destabilize a subset of maternal mRNAs [12,13]. Thus, the role of Smaug in transcript destabilization and the MZT is both direct and indirect.

Related mechanisms appear to implement the MZT in other animals: both maternally encoded RBPs (for example, AU-rich-element binding proteins (ARE-BPs) such as Mex-5/-6 in *Caenorhabditis elegans*; ARE-BP and EDEN-BP in *Xenopus*) and zygotically synthesized *miR*s (for example, *miR-430 *in zebrafish, *miR-427 *in *Xenopus*) function in transcript clearance [14-19].

The above-described studies - both single-gene and genome-wide - have focused on the MZT in the soma, which comprises the vast majority of the embryo. It has been known for some time, however, that primordial germ cells (PGCs) behave very differently from somatic cells in early embryos. For example, in both *Drosophila *and *C. elegans*, PGC formation is directed by localized determinants and their PGCs are transcriptionally repressed when they form (reviewed in [20,21]). The rarity of PGCs in early embryos has largely precluded genome-wide definition of their MZT. However, using green fluorescent protein (GFP)-labeled PGCs and flow cytometry, it is possible to isolate *Drosophila *PGCs [22]. These have been used to analyze enrichment for maternally encoded transcription factors in the PGCs [23] but, to date, no genome-wide analysis of the PGC MZT has been reported.

Here we have used flow cytometry to purify *Drosophila *PGCs from staged wild-type and *smaug*-mutant embryos. A combination of proteomic analyses and comparison with existing whole-mount RNA tissue *in situ *hybridization databases was used to verify successful purification of the PGCs. We then used microarray-based gene-expression profiling to identify maternal mRNAs that are eliminated during the PGC MZT as well as transcripts that are produced during zygotic genome activation in the PGCs. Our data provide the first genome-wide analysis of the PGC MZT and reveal that Smaug regulates both maternal transcript elimination and zygotic genome activation in PGCs.

## Results

### Purified PGCs are enriched for germ-plasm components and ribosomal proteins

**Figure 1:**
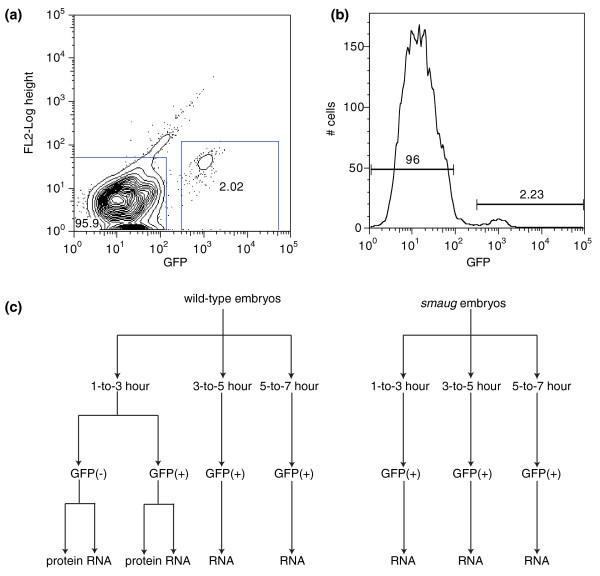
**Flow cytometry and flowchart of experiments**. **(a) **GFP-positive (GFP(+)) and GFP-negative (GFP(-)) cells were obtained by flow cytometric sorting (see Materials and methods). GFP-positive cells are ones with high GFP fluorescence while the GFP-negative cells have low GFP fluorescence. GFP fluorescence is shown on the x-axis (log scale) and FL2 (575 nm band pass sensor; to discriminate dead cells, which autofluoresce) on the y-axis (log scale). The GFP-negative (10^0 ^to 10^2^) and GFP-positive (2 × 10^2 ^to 4 × 10^4^) cells are boxed in blue. **(b) **GFP fluorescence is shown on the x-axis (log scale) and the number of cells in each fluorescence class on the y-axis (linear scale). The GFP-negative cells comprise 96% of the total while the GFP-positive cells comprise 2.23% (horizontal bars indicate the cells that fall into each class). A representative fluorescence activated cell sorting (FACS) run is shown. **(c) **Flowchart of the experiments presented in this study. Protein and RNA levels in the cells were measured by MuDPIT and microarray gene expression profiling, respectively.

**Figure 2:**
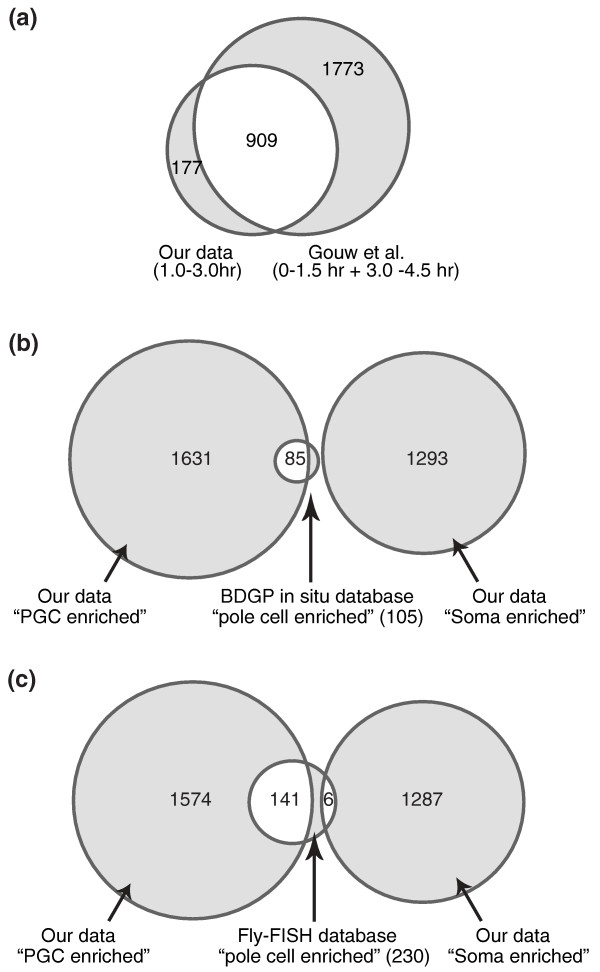
**Venn diagram comparing our lists of proteins and RNAs to previous studies**. **(a) **Venn diagram showing proteins detected in 1-to-3 hour soma and PGCs that have previously been reported to be present in 0-to-1.5 and 3-to-4.5 hour old fly embryos [27]. **(b) **Venn diagram showing overlap between our PGC-enriched and soma-enriched transcripts relative to ones previously reported as 'pole cell localized at stages 4 to 6' in the BDGP *in situ *database [36] (105 genes in total). **(c) **As in (b), but comparing our transcripts with those annotated as 'pole cell enriched at stages 4 to 5' in the Fly-FISH database [37] (230 genes in total). The PGC-enriched and soma-enriched transcripts were determined by comparing the expression profiles of 1-to-3 hour GFP-positive and GFP-negative cells (Additional file 9).

To isolate PGCs from *Drosophila *embryos we modified a published method for flow cytometric sorting of GFP-labeled pole cells [22]; in parallel we sorted GFP-negative cells, which represent the somatic cells (Figure 1). We then used multidimensional protein identification technology (MuDPIT) [24-26] to identify proteins in both the GFP-positive and GFP-negative cells, leading to the identification of a total of 1,086 proteins (Additional files 1 to 3). We compared this list of proteins to 2,711 identified previously as being present in whole early embryos [27]. Of our identified proteins, 909 (84%) were included in their 'whole embryo' list, likely representing proteins that are present in both the bulk cytoplasm and in PGCs and/or at high enough levels in the PGCs to have exceeded the threshold for their whole embryo study (Figure 2; Additional files 4 and 5).

On the basis of spectral counts, 56 proteins were specific to, while 31 were highly enriched in, the GFP-negative cells; these will be referred to here as 'soma-specific' and 'soma-enriched' proteins, respectively (Additional files 6 and 7). The soma-specific list was not enriched for any Gene Ontology (GO) term categories and the soma-enriched list was enriched for a single GO term (Section 1 in Additional file 4).

**Figure 3:**
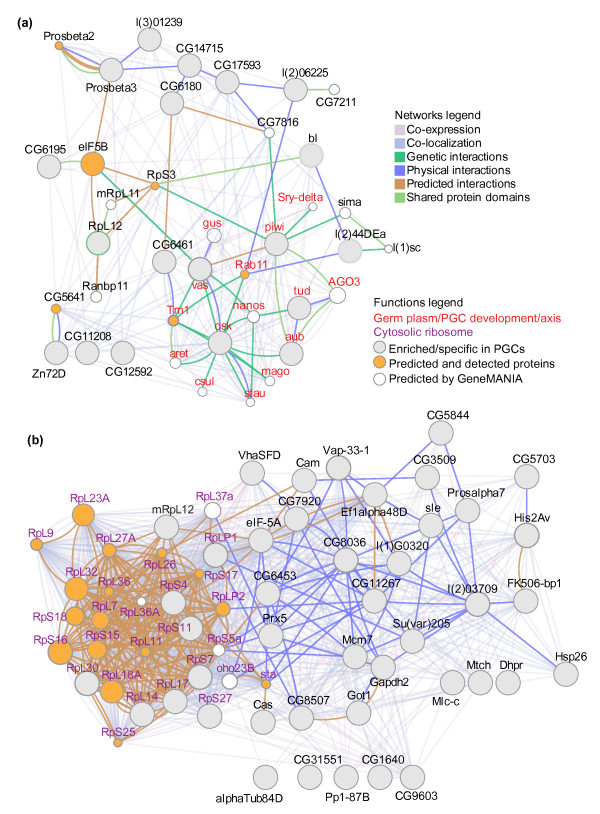
**The PGC proteome**. **(a) **A GeneMANIA-generated network seeded with the proteins specific to PGCs at 1-to-3 hours and linked to the most relevant 20 proteins predicted by GeneMANIA. **(b) **A GeneMANIA-generated network seeded with the proteins enriched in PGCs at 1-to-3 hours and linked to the most relevant 20 proteins predicted by GeneMANIA. The PGC-specific/enriched proteins are gray-filled circles. Proteins that function in germ plasm and/or PGC development are labeled in red. In each case the 20 most relevant predicted proteins are white-filled circles (if they were not detected by our MuDPIT analysis) or orange-filled (if they were detected by our MuDPIT analysis). The predictions of GeneMANIA were based on co-expression, co-localization, genetic interactions, physical interactions, predicted interactions, and shared protein domains [90]. All the detected proteins had a unique peptide number larger than two in the results from mass spectrometry.

Nineteen proteins were specific to, while 43 were highly enriched in, the GFP-positive cells; these will be referred to here as 'PGC-specific' or 'PGC-enriched' proteins, respectively (Additional file 8). GO term analysis of the PGC-specific list showed enrichment for proteins related to germ-plasm assembly and PGC formation, verifying that the GFP-positive cells were indeed PGCs (Figure 3; Additional file 8). The PGC-enriched list was enriched for the GO term 'ribosome'.

Of the 62 PGC-specific and -enriched proteins, a literature review uncovered published immunostain data for seven, six of which were also reported to be PGC-enriched in those data: Tudor, Piwi, Oskar, Vasa, Aubergine, and alpha-Tubulin84D [28-33]. The fact that 6 out of 7 proteins were verified as being present at high levels in the PGCs relative to the soma lends confidence to the remaining 55 also being present at high levels in PGCs (Section 1 in Additional file 4). The verified PGC-enriched/specific proteins are largely involved in the assembly of, and posttranscriptional control in, the germ plasm, while the newly identified but as yet unverified ones included RBPs, protein phosphatases, components of the DNA replication machinery, proteasome subunits, and ribosomal proteins (Figure 3; Section 1 in Additional file 4).

GeneMANIA was used to produce interaction networks by predicting an additional 'top-20' related proteins on the basis of their genetic interaction, physical interaction, co-expression or co-localization with the identified PGC-specific/enriched proteins (Figure 3; Materials and methods). Consistent with the GO term results, the largest GeneMANIA-predicted cluster of PGC-specific proteins comprised germ-plasm components while that for PGC-enriched proteins comprised ribosomal proteins (Figure 3; Additional file 8). We assessed whether any of the other GeneMANIA-predicted proteins were present on our MuDPIT-derived lists of PGC proteins (Additional file 3): 16 out of 20 of the predicted PGC-enriched-protein interactors and 6 out of 20 of the predicted PGC-specific-protein interactors were identified as present (these are shaded orange in Figure 3). GeneMANIA's lower 'success rate' for predicting PGC-specific interactors may derive from the fact that several of these function in the germ plasm earlier, during oogenesis, and are, in fact, not present in the PGCs (for example, Arrest/Bruno and Staufen) [34,35].

### PGC-enriched mRNAs encode germ plasm, stem cell proliferation regulators, DNA damage checkpoints and metabolic enzymes

We next isolated total RNA from the GFP-positive (PGCs) and GFP-negative (somatic cells) sorted from 1- to 3-hour-old wild-type embryos and used these to interrogate microarrays (Figure 1; Additional file 2). We identified 5,695 mRNAs as present in the PGCs and 5,622 as present in the somatic cells (Additional file 9). We identified 1,715 mRNAs as enriched in PGCs and 1,293 as enriched in somatic cells (Additional file 10).

To validate our lists of PGC-enriched and soma-enriched mRNAs, we compared them to lists of transcripts annotated as 'pole cell enriched/localized' in genome-wide RNA tissue *in situ *hybridization databases. Our PGC-enriched transcript list contained 81% (85/105) of the Berkeley Drosophila Genome Project (BDGP) list [36] and 61% (141/230) of the Fly-FISH list [37] (Figure 2; Section 2 in Additional file 4). Importantly, when our somatic-cell-enriched transcript list was compared with the 'pole cell localized' lists in BDGP and Fly-FISH, there was almost no overlap (0% with BDGP and 3% with Fly-FISH). Thus, assuming that all annotations in the BDGP and Fly-FISH databases are correct, we can estimate our true-positive rate as ranging from 61 to 81% and our false-positive rate from 0 to 3%.

The GO terms for somatic-cell-enriched mRNAs related largely to development, cell fate and morphogenesis, and included many transcription factors and cell-cell signaling proteins that are well known to participate in specification of the body axis, cell position and fate (Section 2 in Additional file 4; Additional file 11). For the PGC-enriched transcripts, many of the significant GO terms related to germ plasm, the meiotic cell cycle, metabolism and energy production (Section 2 in Additional file 4; Additional file 11). PGC enrichment for transcripts of germ-plasm proteins is consistent with the fact that many of these mRNAs are known to be localized to the germ plasm and PGCs. The transcripts listed under the 'meiotic cell cycle' GO term included ones that encode proteins that regulate stem cell maintenance and proliferation, and lead to uncontrolled proliferation when mutated (for example, Bag of marbles (Bam), Benign gonial cell neoplasm (Bgcn), Mei-P26, and the tumor suppressor BRCA2) as well as ones that are involved in DNA-damage checkpoints and response (for example, Mei-9, Mei-S332, Mus81, Mus304, Mus309, Mus301, Rad50). Enrichment for transcripts encoding Bam, Bgcn and Mei-P26, which are known to restrict proliferation of germ-line stem cells [38-41], presumably reflects the need to maintain stringent control over PGC proliferation. Enrichment for these transcripts may also reflect an early role in PGCs for factors that function later in development, for example, in stem-cell renewal and/or differentiation. Mei-P26 is also known to repress microRNA activity [41]. Enrichment for transcripts encoding DNA damage checkpoint/repair components is likely to represent an enhanced need to protect against DNA damage in PGCs, thus preventing the germ-line DNA from suffering insults that might affect the ability to produce robust gametes and offspring.

There was also very high enrichment for GO terms related to metabolism and energy production. For example, transcripts encoding seven of the ten enzymes that carry out glycolysis were enriched in PGCs (hexokinase, phosphofructokinase, triosephosphate isomerase, glyceraldehyde phosphate dehydrogenase, phosphoglycerate mutase, enolase, pyruvate kinase). Transcripts encoding alternative pathways for generation of acetyl-CoA for entry into the tricarboxylic acid cycle and respiration were also enriched in the PGCs, as were ones encoding components of fatty acid and amino acid metabolism. Together these data suggest that the PGCs are highly metabolically active when they form. Consistent with this possibility, it has previously been reported that the mitochondria in the germ plasm and PGCs exhibit higher membrane potential than those in the soma, reflective of higher metabolic activity [42].

The mRNA and protein composition of PGCs do not correlate (Additional file 12). This is exactly what might be expected from the fact that maternal proteins may be loaded into the PGCs but their mRNAs specifically excluded (for example, ribosomal proteins versus ribosomal protein mRNAs; see below) while, for other proteins, their maternal mRNAs are loaded into PGCs but not translated (for example, *Cyclin B *mRNA) [43]. In other words, all possible relationships between mRNA and protein levels occur in the PGCs.

### mRNAs that encode germ-plasm components and cell-cell signaling molecules are rapidly degraded during the PGC MZT

**Figure 4:**
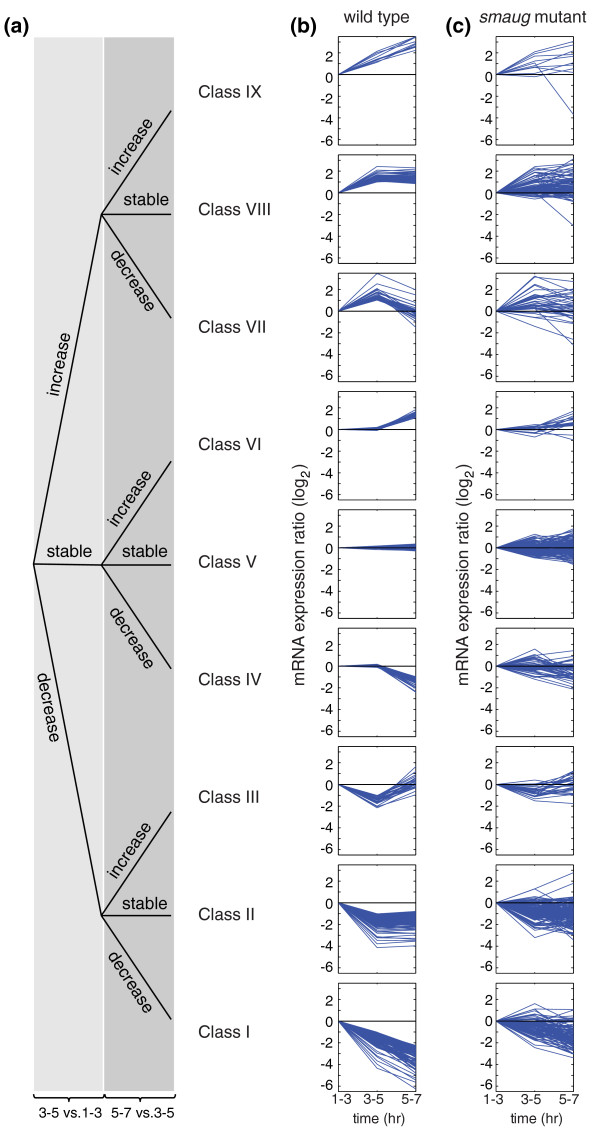
**Classes of transcript profiles during the MZT in PGCs**. **(a) **Decision tree defining the different transcript classes. Expression profiles of transcripts in wild-type PGCs. Class I: transcripts that decrease in level at both the 3-to-5 hour and the 5-to-7 hour time points. Class II: transcripts that decrease in level at 3-to-5 hour but then do not change in abundance at the 5-to-7 hour time point. Class III: transcripts that decrease in level at 3-to-5 hour but then increase in level at the 5-to-7 hour time point. Class IV: transcripts that are present at the same level at the 1-to-3 and 3-to-5 hour time points, but then decrease at the 5-to-7 hour time point. Class V: transcripts that are present at the same level throughout the time course. Class VI: transcripts that are present at the same level at 1-to-3 and 3-to-5 hours, but increase in level at the 5-to-7 hour time point. Class VII: transcripts that increase in level at 3-to-5 hours but then decrease in level at the 5-to-7 hour time point. Class VIII: transcripts that increase in level at 3-to-5 hours, and then remain at the same level at the 5-to-7 hour time point. Class XI: transcripts that increase in level at both the 3-to-5 and 5-to-7 hour time points. **(b) **Expression profiles in wild-type PGCs of the transcripts in these nine classes across the 1-to-3, 3-to-5 and 5-to-7 hour time points. **(c) **Expression profiles of transcripts in Classes I to IX in *smaug*-mutant PGCs. In (b, c) each line represents the average expression profile of a transcript from all the replicates.

To assess changes in mRNA populations during the PGC MZT, we also purified PGCs from 3- to 5- and 5- to 7-hour-old wild-type embryos and used these for microarray-based gene expression profiling (Figure 1; Additional files 2 and 9). We identified mRNAs whose abundance decreased greater than two-fold during this time-course: 810 mRNAs were significantly degraded at 3-to-5 hours and 506 at 5-to-7 hours (Section 3 in Additional file 4; Additional files 13 and 14). Since 99 decreased at both time points, overall, 1,217 of 5,695 (21%) of the mRNAs present in 1- to 3-hour-old PGCs are significantly degraded by 7 hours. We grouped transcripts into nine classes based on the profiles of their elimination and/or production during the time course; five of these classes represent transcripts that undergo clearance with distinct kinetics or timing (Figure 4, classes I, II, III, IV, VII; Additional files 14 to 16; Section 3 in Additional file 4).

GO terms enriched in transcripts destabilized at 3-to-5 hours included several related to posttranscriptional events during assembly and function of the germ plasm (Additional file 17; Section 3 in Additional file 4), consistent with the hypothesis that once PGCs are formed, they transition from a primarily posttranscriptional to a transcriptional mode of gene expression. Of particular interest in this regard is elimination of mRNAs encoding Oskar, which nucleates and directs assembly of the germ plasm; germ cell-less (Gcl), which is required for budding of PGCs (but not germ plasm assembly *per se*); and Orb, a homolog of vertebrate CPEB (cytoplasmic polyadenylation element binding protein), which directs cytoplasmic polyadenylation of mRNAs.

Additional enriched GO terms were for transcripts encoding transmembrane proteins that are likely to be involved in cell-cell adhesion and signaling (for example, Delta, Star, Protein tyrosine phosphatase 10B, Insulin-like receptor, Synaptobrevin, Syndecan, and Wunen). Downregulation of cell-cell adhesion and of specific signaling pathways may be a pre-requisite for subsequent directed migration of the PGCs through the midgut epithelium to the somatic gonad. For example, Wunen is known to be expressed in the midgut and nervous system and to repel germ cells, thus directing them towards the lateral mesoderm [44-47]. Downregulation of Wunen in the PGCs may therefore be required to prevent them from repelling each other as well as to allow expression in the gut and nervous system to provide positional information for their migration. Downregulation of Wunen expression in PGCs may also be required for their survival [45-47].

Significantly enriched GO terms for the five different patterns of decay were largely non-overlapping (Additional file 18; Section 3 in Additional file 4). For example, transcripts that decreased at both time points (class I) were enriched for 'integral to membrane' and 'N-linked glycosylation' GO terms, while those that decreased at the first time point but then were maintained at steady-state levels (class II) were enriched for 'lipid metabolism' and 'FAD binding'. Among the transcripts enriched in class I are ones encoding transmembrane proteins that mediate cell-cell communication, adhesion or fusion (for example, Moladietz, Dual oxidase, Rolling stone, Protein tyrosine phosphatase 10D) [48-52], consistent with a need for ongoing modulation of cell-cell interactions. One of the most interesting transcripts in the FAD-binding category encodes Hermansky-Pudlak syndrome 4 ortholog (dHPS4), an endosomal pathway factor that has recently been implicated in regulation of small interfering RNA loading onto the RNA-induced silencing complex for chromatin silencing [53].

In summary, subsets of the transcripts loaded into the PGCs when they form subsequently exhibit several patterns of instability. The different stability classes are enriched for transcripts encoding proteins with distinct biological, cellular and molecular functions. These observations suggest coordinate regulation - at the level of transcript destabilization - of distinct biological processes in the PGCs.

### Zygotic transcription in PGCs produces mRNAs required for transcription and protein synthesis

We next identified mRNAs that increased greater than two-fold over the time course and therefore must have been transcribed zygotically in PGCs; 657 transcripts increased significantly in abundance in 3-to-5 hour PGCs relative to 1-to-3 hours while 167 increased at 5-to-7 relative to 3-to-5 hours (Additional files 13 and 19; Section 4 in Additional file 4). Thus, zygotic genome activation in PGCs occurs as early as 3-to-5 hours after fertilization (that is, 1.5-to-3.5 hours after the PGCs bud), consistent with the fact that phosphorylation of Ser2 of the RNA polymerase II (Pol II) carboxy-terminal domain (CTD) begins to appear in the nuclei of PGCs 3 hours after fertilization [54].

Zygotically synthesized PGC transcripts were enriched for GO terms related to transcription and ribosomes/translation (Additional file 20; Section 4 in Additional file 4). The newly synthesized mRNAs that fell under the 'transcription' GO term largely encoded core components of the Pol II transcriptional machinery (subunits of Pol II itself, TATA-binding protein (TBP)-associated factors, subunits of the Mediator complex, other factors that associate with Mediator, and chromatin remodeling complexes), consistent with a requirement to produce these factors in order to up-regulate zygotic genome activation in the PGCs.

Regarding ribosomes, our MuDPIT analysis showed that ribosomal proteins are enriched in the PGCs when they form (Figure 3). However, a review of both the BDGP and Fly-FISH databases showed that maternal mRNAs encoding ribosomal proteins are excluded from the PGCs. Zygotic transcription of ribosomal protein mRNAs is, therefore, essential for production of new ribosomal proteins, and thus additional ribosomes, in the PGCs (see Discussion).

Zygotic transcripts could be subdivided into five patterns of production during the time course (Figure 4, classes III, VI, VII, VIII, IX; Section 4 in Additional file 4). As was the case for degraded maternal transcripts in PGCs, GO term analysis indicated that the different classes of zygotic transcripts encoded proteins with distinct biological, cellular and molecular functions (Additional file 20; Section 4 in Additional file 4), suggesting coordinate regulation of different biological processes in PGCs at the level of new transcription. For example, transcripts that increased at 3-to-5 hours and then were maintained at constant levels (class VIII) were enriched for GO terms related to Pol II directed transcription (discussed above) as well as the spliceosome, including the SR protein B52 [55] and a Lsm homolog, Lsm3 [56,57]. These results are consistent with a need for coordinate up-regulation of both primary transcript production and splicing in order to produce mature mRNAs during zygotic genome activation. In contrast, transcripts that increased at both time points (class IX) were enriched for 'male gamete generation'. These transcripts all derive from within, or near to, the Stellate gene cluster (Ste12DOR, Ste:CG33236, Ste:CG33246) that functions in maintenance of male fertility via an RNA interference-related process [58]. *Ste12DOR *transcripts have been reported to be chromatin-associated in early embryos [59].

### Smaug eliminates PGC transcripts encoding proteins that regulate stem cell division, and transcriptional and posttranscriptional regulators

**Figure 5:**
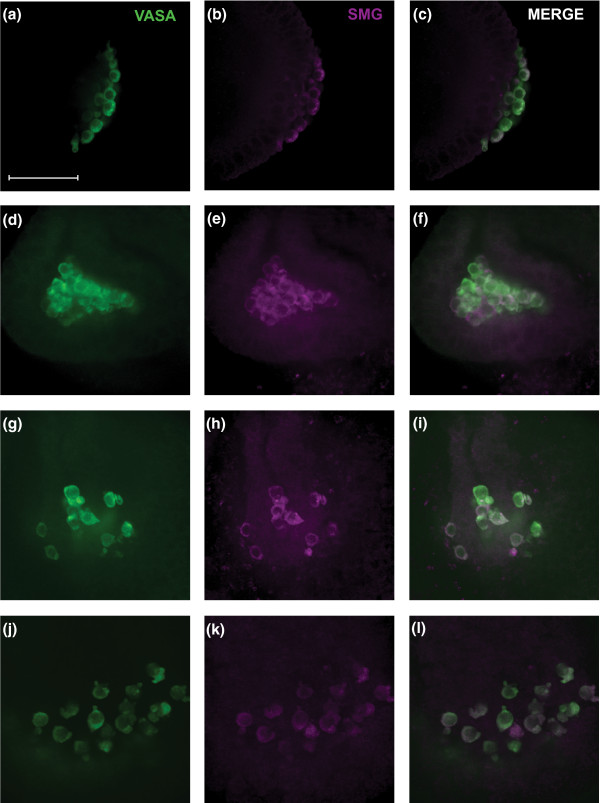
**Smaug protein persists in PGCs**. Double immunostains of Vasa, a PGC marker, and Smaug in PGCs. **(a-c) **Smaug is enriched in PGCs when they form during developmental stage 4, 1.5 hours after fertilization. **(d-f) **Smaug persists in the PGCs at stages 9 and 10, as they sit in the midgut pocket 3-to-5 hours after fertilization. **(g-i) **Smaug persists as the PGCs migrate through the midgut epithelium at stage 10. **(j-l) **Smaug is still detectable at stage 11, 5-to-7 hours after fertilization, at which time the PGCs lie dorsally between the midgut epithelium and the overlying mesoderm. SMG, Smaug protein.

We previously reported that the RNA-binding protein Smaug regulates maternal transcript elimination from the somatic region of the early embryo [3]. Smaug is translated in early embryos and then rapidly degraded after nuclear cycle 13, between 2 and 3 hours after fertilization [3,12]. Smaug protein has been reported to be either uniformly distributed in early embryos [60] or enriched in the germ plasm at the posterior pole and taken up into the PGCs when they bud [61]. To assess the fate of Smaug protein in PGCs, we used immunostaining and confocal microscopy to follow Smaug protein in embryos over the time course of our studies of the PGC MZT. Although Smaug is degraded in the soma, it remains in the PGCs after they bud and throughout the time course (Figure 5).

**Figure 6:**
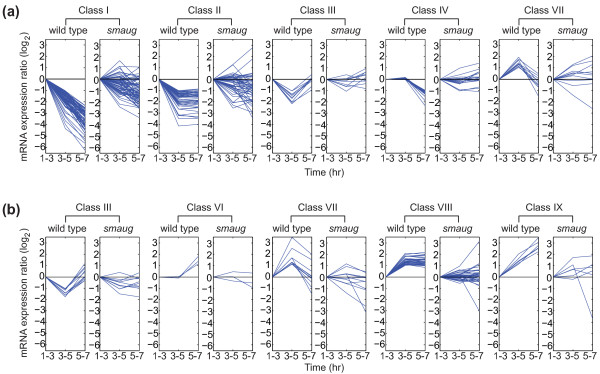
**Smaug-dependent RNA decay and/or transcription of class I to IX transcripts**. **(a) **Smaug-dependent RNA decay. Class I: transcripts stabilized at either 3-to-5 or 5-to-7 hours. Class II: transcripts stabilized at 3-to-5 hours. Class III: transcripts stabilized at 3-to-5 hours. Class IV: transcripts stabilized at 5-to-7 hours. Class VII: transcripts stabilized at 5-to-7 hours. **(b) **Smaug-dependent zygotic transcription. Class III: transcripts that fail to increase at 5-to-7 hours. Class VI: transcripts that fail to increase at 5-to-7 hours. Class VII: transcripts that fail to increase at 3-to-5 hours. Class VIII: transcripts that fail to increase at 3-to-5 hours. Class XI: transcripts that fail to increase either at 3-to-5 or 5-to-7 hours. Each line represents the average expression profile of a transcript from all the replicates. Each pair of plots represents the expression profiles of the same group of Smaug-dependent transcripts in wild-type (left panel) and *smaug*-mutant PGCs (right panel).

Given the persistence of Smaug in PGCs, we decided to assess whether Smaug regulates the MZT in PGCs. We purified GFP-labeled PGCs from 1- to 3-, 3- to 5- and 5- to 7-hour-old embryos produced by *smaug *hemizygous females. We then used RNA isolated from the sorted cells to interrogate microarrays and identified transcripts greater than two-fold enriched (or depleted - see next section) in mutant relative to wild-type PGCs (Figures 1, 4 and 6). Of the 810 mRNAs that decreased significantly in wild-type 3-to-5 hour PGCs, 301 (37%) were significantly stabilized in *smaug *mutant PGCs while 142 (28%) of the 506 that decreased in wild-type 5-to-7 hour PGCs were stabilized in the mutant (Additional file 21). Overall, 34% of unstable PGC transcripts were Smaug-dependent. To verify the list of Smaug-dependent PGC transcripts, we selected several for fluorescence *in situ *hybridization, the results of which confirmed that they were stabilized in *smaug *mutant PGCs relative to wild type (Additional file 22).

GO term analysis indicated that PGC transcripts dependent on Smaug for degradation were enriched for terms related to posttranscriptional regulators of germ plasm assembly, control of stem cell division, transcriptional regulation, and developmental proteins (Additional file 23; Section 5 in Additional file 4). The first of these terms was also enriched in all unstable mRNAs (and is discussed above); the Smaug-dependent transcripts related to posttranscriptional regulators included those encoding Aret/Bruno, BicC, Exu, and Smaug itself.

The 'transcriptional regulation' and 'developmental protein' GO terms were only enriched in the Smaug-dependent subset of transcripts (that is, not enriched in all unstable transcripts). These included transcripts encoding transcription factors such as GAGA factor (Trithorax-like) and a negative regulator of GAGA, Tramtrack (see Discussion); Lola, a zinc-finger/BTB-containing transcription factor required for self-renewal in neural stem cells [62] and also recently implicated in gonad assembly in the embryo [63]; FOXO and InR (the *Drosophila *insulin receptor), which are key components of insulin-directed regulation of cell number [64,65]; Numb, a regulator of Notch signaling that is known to be expressed in stem cells and to regulate asymmetric cell fate specification [48,66]; and Kokopelli, a cyclin required for germ-line stem cell renewal (JD Baker and MJ Kernan, personal communication). It is noteworthy that additional PGC-enriched transcripts that control cell proliferation and stem cell divisions are also Smaug-dependent for elimination (for example, BRCA2 at 3-to-5 hours, Bam at 5-to-7 hours). Thus, Smaug is likely to regulate stem-cell-like features of PGCs, including their proliferation.

### Smaug is required for zygotic genome activation in PGCs

We previously showed that, in *smaug *mutants, high-level zygotic genome activation fails in the somatic region of the embryo and provided data suggesting that this is an indirect effect of misregulation of maternal mRNAs that keep the genome silent in early embryos [12]. To assess whether Smaug also regulates zygotic genome activation in the PGCs, we compared zygotically expressed transcripts in wild type versus *smaug *mutants (Figures 1, 4 and 6). Overall, 36% of zygotically expressed transcripts were expressed at significantly lower levels in the mutant PGCs: 248 (38%) of 657 transcripts that increased in abundance in wild-type PGCs at 3-to-5 hours, and 50 (30%) of 167 transcripts that increased at 5-to-7 hours (Additional file 24).

When the Smaug-dependent lists were compared to all genes on the array, the enriched GO terms substantially overlapped those enriched in wild type (Additional file 25). There were no significantly enriched GO terms for either of the Smaug-dependent lists relative to the lists of zygotic transcripts at these time points. Furthermore, none of the subclasses of affected mRNAs yielded significantly enriched GO terms. Together, these results are consistent with Smaug being required indirectly for zygotic genome activation in PGCs.

### Transcripts that are Smaug-dependent for decay are enriched for SREs while those that are Smaug-dependent for transcription are depleted for SREs

**Figure 7:**
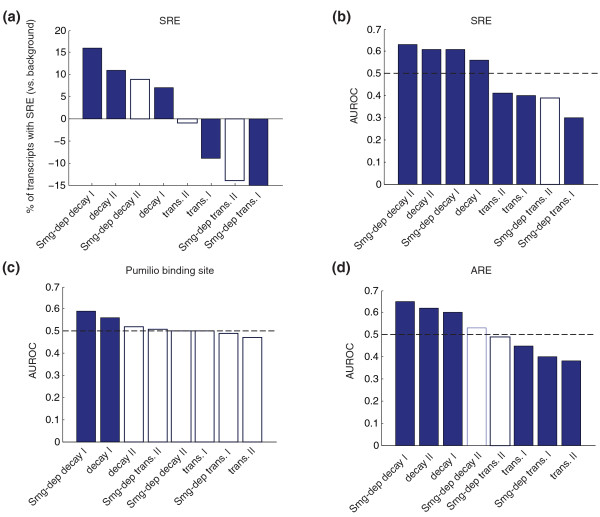
**Enrichment of RBP binding sites in PGC transcripts**. **(a) **Enrichment of SREs evaluated by comparing the percentage of the transcripts having at least one SRE (that is, CNGGN_(0-3) _loop sequence with a four base pair stem) in the specific transcript category, relative to the background set (that is, all transcripts expressed at the same time point). Significance of enrichment was assessed by Bonferroni-corrected hypergeometric *P*-values. **(b) **Enrichment of SREs tested by comparing accessibility and the presence of CNGG in the specific category (that is, the positive set) versus the control category (that is, the negative set, which contained the transcripts expressed at the same time point but without any change in the expression level). Area under the receiver operator characteristic (AUROC) and Bonferroni-corrected Wilcoxon-Mann-Whitney rank sum *P*-values were used to represent the enrichment results and the significance level. AUROC equal to 0.5 (the dashed line), larger than 0.5 or smaller than 0.5 separately indicate that the binding site is represented equal to, more, or less in the positive set versus the negative set. **(c, d) **Graphs similar to (b), but representing the enrichment results for the Pumilio-binding-site (c) and AU-rich element (ARE) (d). Results that pass the significance test are shown with filled bars (multiple-test-corrected *P*-value ≤0.05) and the non-significant results as unfilled empty bars (multiple-test-corrected *P*-value > 0.05). Decay or transcription ('trans.') at the 3-to-5 hour time point relative to 1-to-3 (I) or at the 5-to-7 hour time point relative to 3-to-5 (II). *smg, smaug *mutant.

Direct targets of Smaug should carry one or more SREs while indirect targets need not contain SREs. We therefore assessed the different classes of transcripts for SREs using methods that we developed previously [3,67]. Consistent with a direct role for Smaug in PGC transcript destabilization, both unstable and Smaug-dependent unstable transcripts exhibited enrichment for SREs (Figure 7). In contrast, zygotically produced PGC transcripts and Smaug-dependent zygotic mRNAs did not show enrichment for SREs; indeed, SREs were depleted in these classes of transcripts (Figure 7).

The depletion of SREs among Smaug-dependent zygotic transcripts is consistent with Smaug's effect on zygotic genome activation in PGCs being indirect. Furthermore, depletion for SREs suggests that, in order for zygotically synthesized transcripts to remain stable in the presence of Smaug protein, there may have been selection against SRE-like elements.

### Additional RBP- and *miR*-binding sites are enriched in unstable PGC transcripts

We next assessed whether there was enrichment (or depletion) of target sites for additional RBPs that are known to be expressed in early embryos (Pumilio [68]) and/or that have previously been shown to have target-site enrichment among maternal transcripts in early embryos (Pumilio and ARE-BPs [69]). Pumilio-binding sites were enriched only in PGC-transcripts that are degraded at the 3-to-5 hour time point (Figure 7), suggesting a role during the initial phase of transcript destabilization in PGCs. Unexpectedly, AREs showed a similar distribution to SREs: enrichment in unstable PGC transcripts and depletion in zygotic PGC transcripts (Figure 7). However, AREs and SREs are not co-enriched and co-depleted in the same transcripts. For example, among 738 PGC transcripts that are destabilized at 3-to-5 hours of embryogenesis, 51% have SREs, 51% have AREs and 25% have both SREs and AREs (26% is expected on the basis of independence). These results suggest that Smaug and ARE-BPs function independently in transcript destabilization during the PGC MZT.

Pumilio protein is present in the PGCs (AL Goldman and HDL, unpublished observation). It should be noted, however, that there is no published evidence that ARE-BPs are present in PGCs. From our MuDPIT analyses, 4 of the 11 predicted ARE-BPs may be expressed in early embryos: one, P element somatic inhibitor (Psi), was present on our soma-specific list; two (Rox8 and CG8778) were ambiguous since they were present only in one of the two replicates; and one, Hrb27C/Hrp48/p50, was present at high levels in both soma and PGCs. This last ARE-BP is known to function in regulation of translation in the soma of early embryos [70].

We next assessed *miR *target site enrichment in the different PGC transcript stability classes using Pictar and TargetScan [71,72]. Unstable PGC transcripts were enriched for several *miR *target sites (Additional file 26), including those for the *miR-309 *cluster, which is transcribed in a Smaug-dependent manner in early embryos and has previously been shown to target a subset of maternal transcripts for elimination [12,13]. Whether a particular *miR *is in fact able to destabilize its targets is dependent on whether it is present at the right time and place to do so. For example, although the *miR-309 *cluster is transcribed at high levels in early embryos [12,13], available *in situ *hybridization data show that *miR-309 *transcription is restricted to the somatic region and does not occur in the PGCs [73]. Thus, *miR-309 *cannot destabilize its targets in the PGCs. Likewise, although unstable PGC transcripts are enriched for *miR-1, miR-2a-2 *cluster, *miR-8, miR-10, miR-11, miR-13b-1 *cluster, *miR-92a, miR-274*, and *miR-283 *target sites, all of these *miRs *are expressed in the soma but not in the PGCs [73]. These results suggest a mechanism that explains why the MZT is delayed in PGCs relative to the soma (see Discussion).

### Comparison of the MZT in the soma and PGCs

A comparison of the MZT in the soma versus the germ line is shown in Table 1. The scale of the MZT in the PGCs and soma is similar: in the soma 1,600 to 2,100 maternal transcripts are eliminated [3,69] while we have shown here that about 1,300 transcripts are eliminated from the PGCs. Likewise, zygotic genome activation produces 900 to 1,000 transcripts in the soma [12,69] and about 800 in the PGCs.

**Table 1:** Comparison of MZT in the soma versus the PGCs

	Soma		Primordial germ cells	
**RNA decay**				
Material analyzed	Unfertilized egg [3]	Embryo [69]	PGCs (this study)	PGCs (this study)
Time	2-to-4 hours, 4-to-6 hours versus 0-to-2 hours	2-to-3 hours versus 0-to-1 hours	3-to-5 hours versus 1-to-3 hours	5-to-7 hours versus 3-to-5 hours
Gene numbers	1,637	2,107	810	506
Genes dependent on Smaug for RNA decay	975 (60% of RNA decay)	NA	301 (37% of RNA decay)	142 (28% of RNA decay)
SREs in RNAs expressed	48%	46%	45%	45%
SREs in RNA decay	59%	63%	52%	56%
SREs in Smaug-dependent RNA decay	67%	NA	61%	54%
GO terms for RNA decay	Transmembrane transport Proteasome DNA damage/repair Alternative splicing Cell/developmental maturation Nucleotide-binding	Nucleotide-binding Metabolism germ cell Development Alternative splicing Sexual reproduction	Intrinsic/integral to membrane EGF-like domain Ras/Ras GTPase N-linked glycosylation Pole plasm RNA localization Pole plasm assembly Embryonic axis specification Alternative splicing	Intrinsic/integral to membrane Dephosphorylation Lipid catabolism Alternative splicing
GO terms for Smaug-dependent RNA decay	Cell cycle phosphoprotein Nucleotide-binding Microtubule-based process Chromosome organization Proteasome	NA	Alternative splicing Developmental protein Cell surface receptor-linked signal transduction	Electron carrier activity
**Zygotic transcription**				
Material analyzed	Embryo [69]	Embryo [12]	PGCs (this study)	PGCs (this study)
Time	0-to-1 hours versus 1-to-2 hours	2-to-3 hours versus stage 14 oocytes	3-to-5 hours versus 1-to-3 hours	5-to-7 hours versus 3-to-5 hours
Gene numbers	1,110	939	657	167
Genes dependent on Smaug for zygotic transcription	NA	371 (40%)	248 (38%)	50 (30%)
SREs in zygotic transcription	54%	48%	35%	43%
SREs in Smaug-dependent zygotic transcription	NA	36%	29%	30%
GO terms for zygotic transcription	Transcription Morphogenesis Interphase Development	Developmental protein Morphogenesis transcription Metabolism	Structural constituent of ribosome Structural constituent of mitochondrial ribosome Mitochondrial membrane part mitochondrial electron transport Transcriptional regulator activity	Casein kinase II mitotic spindle organization
GO terms for Smaug-dependent zygotic transcription	NA	Morphogenesis Signal Cell fate	No significant GO terms	No significant GO terms

NA, not available since *smaug *mutants were not analyzed.

The population of transcripts loaded into the PGCs when they bud differs from that in the somatic cells: of the 5,695 transcripts present in PGCs, 838 (15%) are PGC-specific while, of the 5,622 transcripts present in the somatic cells, 765 (14%) are soma-specific; 4,857 transcripts are present in both PGCs and somatic cells. The differences in PGC versus somatic transcript populations are a consequence of three mechanisms that operate during oogenesis and early embryogenesis: transport-based transcript localization to the germ plasm; spatially regulated transcript decay in the soma but not the germ plasm; and active exclusion of transcripts from uptake into the PGCs when they bud.

The differences in transcript populations in the PGCs and soma underlie the differences in GO term enrichment in destabilized maternal mRNAs in the soma versus the PGCs: while certain terms were shared, many were unique to one or other cell type (Table 1). This was particularly evident for the Smaug-dependent unstable transcripts, for which the enriched GO terms did not overlap at all.

As described above, unstable PGC transcripts are enriched for SREs while the newly synthesized PGC transcripts are depleted for SREs. This observation prompted us to reassess maternal and zygotic somatic transcripts that we identified previously [3,12]. In the soma, the trend was similar to that in the PGCs: 67% of Smaug-dependent unstable transcripts had at least one SRE, as did 59% of all unstable transcripts, and 48% of all maternal and all zygotic transcripts, but only 36% of Smaug-dependent zygotic transcripts, had an SRE (Table 1). These results are consistent with the fact that zygotic genome activation in the soma initiates while Smaug protein is still present at high levels; it is only after genome activation that Smaug protein rapidly disappears [12].

Zygotic transcription in the soma and PGCs produces transcripts enriched for largely non-overlapping GO terms (Table 1): in the soma, related mostly to development, morphogenesis and metabolism; in the PGCs largely related to ribosomes and mitochondria. These differences presumably reflect the very different biology of the germ line and soma: for the soma, specification of cell positional identity and assembly of a three-dimensional embryo with multiple cell types and tissues; for the PGCs - which represent a single cell-type - the imperatives of coordinate transcription, translation and energy production.

## Discussion

Here we have defined the *Drosophila *PGC MZT and have identified Smaug as a major regulator of both aspects: maternal transcript elimination and zygotic genome activation. We previously identified these roles for Smaug in the somatic MZT [3,12]. Thus, Smaug regulates the MZT in both the soma and the germ line.

### Maternal mRNA degradation in PGCs

Our genome-wide analysis of PGCs has shown that several classes of transcripts are enriched in PGCs when they form: these encode germ-plasm components, regulators of stem cell maintenance and proliferation, DNA damage checkpoints, and metabolic enzymes. A subset of these is eliminated during the MZT. We do not know whether the proteins encoded by these mRNAs decay with similar kinetics to their mRNAs; however, because a recent study has demonstrated coordination of mRNA and protein turnover in the soma of early *Drosophila *embryos [74], we will, for the purposes of this discussion, assume that there is an overall correlation between RNA and protein production and decay. The fact that unstable transcripts that exhibit distinct types of decay profiles are enriched for distinct GO terms suggests that coordination of different biological processes is regulated at the level of mRNA stability.

Transcripts that are eliminated during the PGC MZT encode posttranscriptional regulators that function in germ-plasm assembly, consistent with a transition to a primarily transcriptional mode of gene regulation; and cell-cell signaling molecules, modulation of which may be essential for directed migration of the PGCs. The fact that transcripts encoding metabolic enzymes are not eliminated is consistent with an ongoing high metabolic requirement in these cells, as evidenced by very high mitochondrial activity [42]. High metabolic function also leads to production of reactive oxygen species that can cause DNA damage, which may be one reason why transcripts encoding DNA damage checkpoints and the DNA repair machinery are maintained at high levels. Protection from damage is a particularly crucial imperative for germ-line DNA since it will ultimately give rise to the next generation.

Smaug targets a smaller proportion of PGC transcripts for elimination than it does in the soma - a third in PGCs versus two-thirds in the soma [3] - but, nevertheless, remains a major regulator of this aspect of the PGC MZT. Enrichment of the Smaug-dependent transcripts for SREs lends confidence to the interpretation that their decay is a direct consequence of binding by Smaug and recruitment of the CCR4/POP2/Not-deadenylase [7,8,67]. Smaug-dependent PGC transcripts include those that encode posttranscriptional regulators of germ-plasm assembly and function, regulators of stem cell proliferation, as well as both positive and negative transcriptional regulators. These results suggest that Smaug plays a major role in specification of PGC fate, proliferation, and regulation of gene expression. Further effort is required to define the cellular consequences of failure to eliminate these classes of transcripts in *smaug *mutants.

### Zygotic genome activation in PGCs

Zygotic transcription in the PGCs produces the core machinery for both transcription and translation: for transcription, Pol I, Pol II and associated factors, as well as the splicing machinery; for translation, ribosomal proteins. This is consistent with a requirement to coordinate production of the RNA- and protein-synthetic machinery in PGCs during zygotic genome activation. In the soma, zygotic genome activation produces both the core transcriptional machinery and a large number of spatially restricted transcription factors that specify cell identity [69]. Thus, in both the PGCs and the soma, zygotic genome activation establishes a positive feedback loop that permits the zygotic genome to establish control of development.

Ribosomal protein synthesis accounts for up to 10% of all protein synthesis in the soma of early *Drosophila *embryos; surprisingly, however, it is only after blastoderm formation that the newly synthesized components are incorporated into ribosomal subunits [75,76]. Synthesis of new ribosomal protein mRNAs in the PGCs is particularly important in light of the fact that maternally loaded mRNAs encoding these proteins are excluded when the PGCs bud. While the biological reasons for this exclusion are unclear, we speculate that production of fresh ribosomes composed of newly synthesized rRNAs and proteins may be required, not just for high-level, but also for high-fidelity protein synthesis in the PGCs, a cell type in which fidelity must be particularly important.

An additional possible reason for synthesis of new ribosomal protein mRNAs relates to the production of specific isoforms to produce distinct 'flavors' of ribosomes [77]. It has been reported that the germ-line stem cells in the *Drosophila *gonads express mRNAs encoding specific isoforms of RpS5, RpS10, RpS19 and RpL22, which might confer the ability to interact with alternative eIF-4F members [78]. However, our analyses indicate that RpS5b mRNA and protein are present at equivalent levels in both the PGCs and the somatic cells; that RpS10a and RpL22L mRNAs and proteins are not detectable in either the PGCs or soma; and that RpS19b mRNA and protein are undetectable in the PGCs and soma of 1-to-5 hour embryos. However, RpS19b transcripts are synthesized in PGCs at 5-to-7 hours, consistent with the possibility that these contribute to production of 'stem cell-like' ribosomes. It will be interesting to determine whether transcripts encoding the other stem cell-like isoforms are induced later, when the PGCs reach the somatic component of the gonads.

### Mechanisms underlying the differential timing of the MZT in PGCs and soma

Elimination of maternal mRNAs from the soma is triggered by egg activation and commences before fertilization [3,79-81]. In contrast, the present study has shown that elimination of maternal mRNAs from PGCs is delayed relative to the soma, commencing about 3 hours after fertilization. In the case of the soma we were able to use stage 14 oocytes as our 'undegraded' reference in which to define all maternally contributed mRNAs [3]. Since the PGCs bud from the posterior pole of the embryo 1.5 hours after fertilization, and up to 70% of maternal transcripts exhibit a non-uniform spatial distribution in embryos [59], we used PGCs sorted from 1-to-3 hour embryos as our reference. Thus, we cannot exclude the possibility that, had we used more and narrower time windows, we would have detected decay of certain PGC transcripts prior to the 3-to-5 hour time point.

Zygotic transcription in the soma initiates in two waves, with a small subset of transcripts being synthesized as soon as 30 minutes after fertilization and high-level zygotic genome activation beginning after 2 hours [59,69,82]. With respect to the current study, the same caveat applies to zygotic genome activation in PGCs as to maternal transcript clearance: certain transcripts may be produced prior to the 3-to-5 hour time point but not be detected in our study. We suspect that this is unlikely because genome-wide *in situ *hybridization analyses of 0- to 3-hour-old embryos have not detected sites of zygotic transcription in PGCs (see the Fly-FISH database). Furthermore, there are mechanisms to ensure that the PGCs are transcriptionally silent when they form [21,83]. Indeed, it has been shown that Pol II CTD Ser2 phosphorylation, a hallmark of transcription elongation, is absent from the PGCs when they form, but begins to appear at 3 hours of embryogenesis [54]. Our data showing that zygotic genome activation in PGCs occurs at 3-to-5 hours of embryogenesis is, thus, fully consistent with the timing of CTD Ser 2 phosphorylation. Taken together, our data on transcript elimination and production suggest that the MZT in PGCs commences later than in the soma.

**Figure 8:**
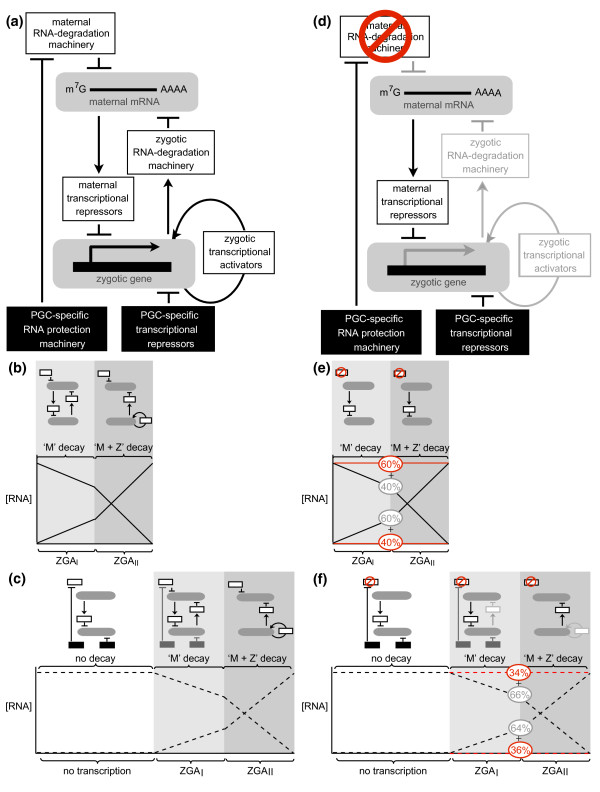
**Model for delayed MZT in the PGCs relative to the soma**. **(a-c) **Wild type, **(d-f) ***smaug *mutant. (a, d) The model. (b, c, e, f) The dynamics of maternal decay and zygotic genome activation with the model diagrammed above the curves. (a, b) In wild-type soma, maternal mRNAs are targeted for decay by a maternally encoded decay machinery ('M') that includes Smaug. Among the targeted transcripts are ones encoding transcriptional repressors that keep the zygotic genome silent. As these are eliminated so zygotic genome activation (ZGA) initiates. Among the zygotic transcripts are components of the zygotically encoded decay machinery ('Z') that feed back to further destabilize maternal mRNAs. ZGA also produces transcriptional activators that feed back to upregulate transcription. (a, c) In wild-type PGCs additional layers of regulation occur (black boxes): protection of a subset of maternal mRNAs from decay factors such as Smaug; and PGC-specific transcriptional repressors that keep the zygotic genome silent (for example, Polar granule component). Only after these are eliminated does the delayed MZT commence in the PGCs. (d, e) In *smaug*-mutant soma, a subset of maternal mRNAs is stabilized (60%) while a subset of ZGA fails (40%). (d, f) In the *smaug*-mutant PGCs, there is a similar effect but on a different scale: 34% of unstable maternal mRNAs fail to be cleared and 36% of ZGA fails.

What is the mechanism underlying the delay in maternal transcript elimination from PGCs? In the soma, while decay initiates upon egg activation, zygotic transcription is required for production (or activation) of more potent decay activities that feed back to further destabilize maternal mRNAs [80] (Figure 8a, b). The zygotically synthesized decay activities are encoded by different chromosomes [69] and include the *miR-309 *family of microRNAs [13]. The PGCs bud before the zygotically encoded decay machineries are synthesized and are transcriptionally silent when they form, so synthesis of *miR-309 *[73] and, likely, other components of the zygotically synthesized machinery is restricted to the soma. Thus, PGCs contain the maternally encoded transcript elimination machinery but not the zygotically encoded decay machinery. We showed previously that the maternal machinery is about one-third as efficient as the combined maternal and zygotic machineries and that many transcripts are protected from this machinery in the PGCs [79,80]. Thus, the elimination of maternal transcripts from PGCs occurs with slower kinetics than in the soma (Figure 8a, c).

In the soma, Smaug regulates mRNA translation and decay, providing specificity to the mechanism by targeting transcripts that contain SREs [4-9]. Smaug is also required indirectly for the production of the zygotically synthesized components of the decay machineries (for example, *miR-309*) [12]. In the germ plasm and PGCs, Smaug's ability to repress translation is abrogated [6,9,84,85] while transcripts that Smaug eliminates from the soma persist [7,8,80] (Figure 8d, e). Persistence of Smaug-dependent transcripts in PGCs may be due, at least in part, to slower decay kinetics caused by the absence of the aforementioned zygotic decay factors (Figure 8d, f).

We have hypothesized that zygotic genome activation in the soma requires Smaug-dependent elimination of maternal RNAs encoding factors that keep the genome silent [12]. A key repressor of the genome in PGCs is the protein encoded by the *Polar granule component *gene [21,70,83]. Our data indicate that *Polar granule component *mRNA is present at high levels in both wild-type and *smaug*-mutant PGCs throughout the time-course of our experiments. Additional factor(s) must, therefore, be eliminated in order to accomplish activation of the PGC genome: one strong candidate is Tramtrack, a well-known repressor of gene activation during the somatic MZT [12,86], whose mRNA is eliminated from the PGCs in a Smaug-dependent fashion.

## Conclusions

PGC-enriched mRNAs encode germ plasm, stem cell proliferation regulators, DNA damage checkpoints and metabolic enzymes. During the PGC MZT, mRNAs encoding germ-plasm and cell-cell signaling molecules are rapidly degraded while zygotic transcription produces mRNAs encoding the core transcriptional and protein synthetic machineries. We identified nine different classes of transcript decay/production profiles and showed that these are enriched for distinct classes of transcripts, suggesting coordinate regulation of different biological processes at the transcriptional and posttranscriptional levels. Smaug is required for elimination of a third of the degraded PGC transcripts, notably ones encoding proteins that regulate aspects of stem cell behavior, and transcriptional and posttranscriptional processes. Computational analyses showed that *cis*-acting SREs as well as AREs are enriched in the degraded transcripts and depleted in newly synthesized transcripts. That SREs and AREs are not co-enriched in the same transcripts suggests independent functions for Smaug and ARE-BPs in the PGC MZT. A comparison of the somatic and PGC MZTs showed that, while the scale of these processes is very similar, the specific transcripts that are eliminated or produced are quite different, reflecting the very different developmental imperatives of these cell types. The PGC MZT is delayed relative to that in the soma, likely because relief of PGC-specific transcriptional silencing is required for zygotic genome activation as well as for efficient maternal transcript clearance.

## Materials and methods

### Fly stocks, maintenance and embryo collection

The following fly strains were used: *w^1118^, VASA-GFP*/*CyO *; *PrDr*/*TM3, Sb *[87], *VASA-GFP*/*CyO *; *smg^1^/TM3, Sb *and *Df(3L)Scf-R6*/*TM3, Sb *(Bloomington Stock #4500). Stock maintenance and embryo collections followed standard procedures that are detailed in Additional file 5.

### Pole cell (PGC) isolation

Pole cells were isolated from the embryos by modifying a previously published protocol [22]. For RNA isolation, embryos dechorionated in a 50% bleach solution were washed and collected on a 125 μm nylon mesh. Small aliquots (approximately 500 embryos) were transferred into a 7 ml Dounce homogenizer on ice containing 500 μl of S2 medium (Schneider's insect medium; Sigma-Aldrich, St Louis, MO, USA). Embryos were gently homogenized using a loose-fitting pestle by turning the pestle 180 degrees, slowly pulling up the pestle to create a gentle shearing force and then allowing it to drop down into the homogenizer on its own weight. This was repeated eight to ten times to allow maximum disruption of the embryos without damaging the pole cells. Embryo extract was collected in a fresh 10 ml plastic tube and kept on ice until all the embryos were processed. The extract was filtered once through a 125 μm nylon mesh to remove large chunks of embryo debris and centrifuged in a hanging-basket rotor at 1,000 rpm for 10 minutes at 4°C. The supernatant was removed and the pellet was gently resuspended by pipetting up and down in 5 ml of ice-cold calcium-free PBS (Life Technologies Corporation, Carlsbad, CA, USA). After centrifugation as described above, the supernatant was discarded and the pellet was gently resuspended in 2 ml of calcium-free PBS containing 0.1% trypsin (Sigma-Aldrich) and was incubated at 37°C for 10 minutes. An equal volume of S2 medium supplemented with 20% fetal bovine serum was added to the extract to terminate the proteolysis. In order to separate the dissociated pole cells from the nucleic acid strands visible after the trypsinization, 10 units of DNase I (Life Technologies Corporation, Carlsbad, CA, USA) was added to the extract, pipetted up and down gently and incubated at room temperature for 15 minutes. The extract was centrifuged as above, the pellet was resuspended in 1 ml of S2 medium, filtered through a 40 μm nylon mesh and stored on ice until cell sorting.

### Fluorescence-activated cell sorting (FACS) of PGCs

Cells were sorted using a BDFACS Aria (BD Biosciences, Franklin Lakes, NJ, USA) flow cytometer fitted with a 100 μm nozzle at 35 psi. Samples were sorted at 4°C and the sorted cells were collected into 1.5 ml of S2 medium at 4°C. Post-sort purity of GFP-positive cells was greater than 98% in the collected samples. The yield for VASA-GFP positive cells ranged from 25,000 to 110,000 cells/sort, starting from 8 cages of flies.

### Mass spectrometry

PGC isolation for mass spectrometry was as above but without trypsin and DNase I treatment. The PBS-washed pellet was resuspended in approximately 1.0 ml PBS and filtered through a 40 μm mesh twice for FACS. The sorted cells were collected into tubes containing 1.5 ml PBS. Post-sorting, cells were centrifuged at 850 × g for 10 minutes at 4°C. The samples were flash frozen and stored at -80°C. The numbers of cells used were: GFP-positive cells, 5.96 × 10^5 ^(replicate 1) and 4.89 × 10^5 ^(replicate 2); GFP-negative cells, 1.3 × 10^6 ^(replicate 1) and 1.0 × 10^6 ^(replicate 2). Details of MuDPIT materials, sample preparation and mass spectrometric analysis are presented in Additional file 7 and followed standard methods [25]. Samples were analyzed on a LTQ-Orbitrap XL instrument. Database searches and protein identification used Sequest Sorcerer™ (version 3.5, Sage-N Research, Milpitas, CA, USA) against a NCBI *Drosophila melanogaster *protein sequence database (14,331 sequences). Validation of tandem mass spectrometry-based peptide and protein identifications was performed using the Scaffold software (version Scaffold 2.1.1, Proteome Software Inc., Portland, OR, USA). Peptide identification was carried out using the Peptide Prophet algorithm [88]. Additional details regarding the protein identification methods are given in Additional file 5.

### Total RNA isolation, amplification and microarray-based gene expression profiling

Post sorting, cells were centrifuged as described above, S2 medium was removed, and 1 ml of TRIzol (Life Technologies Corporation) was added to the cell pellet. Samples were flash frozen in liquid nitrogen and stored at -80°C until further processing. For each biological or technical replicate, 15 to 20 × 10^3 ^cells were thawed at room temperature in TRIzol and total RNA was isolated according to the manufacturer's protocol. RNase-free glycogen (20 μg; Thermo Fisher Scientific Inc., Waltham, MA, USA) was added to each RNA precipitation to improve total RNA yield. The RNA pellet was air-dried and resuspended in 30 μl of RNase-free water. RNA quality and quantity were determined using the Bioanalyzer-RNA Pico kit (Agilent Technologies, Santa Clara, CA, USA). Typical RNA yield ranged from 0.5 to 2 ng/μl.

The WT-OVATION Pico RNA Amplification System (NuGEN Technologies, San Carlos, CA, USA) was used to reverse-transcribe and amplify 5 ng of total RNA from each sample according to the supplier's protocol. The amplification yield ranged from 6.5 to 7.5 μg of single-stranded antisense cDNA. Before fluorescent labeling of cDNA for microarray hybridization, 500 ng of the single-stranded antisense cDNA was used in a standard Klenow reaction using random hexamers (Life Technologies Corporation) to synthesize the second cDNA strand. Double-stranded cDNA product (1 μg) from the Klenow reaction was used for fluorescent labeling using the NimbleGen DNA labeling kit (Roche NimbleGen, Madison, WI, USA) according to the manufacturer's protocol. Cy3- and Cy5-tagged random nonamers (Dual-Color DNA labeling kit, Roche NimbleGen) were used to label cDNA from wild type and *smg^1^*/*Df *PGCs, respectively. Custom-designed *Drosophila *4 × 72K Nimblegen arrays were used and each array was hybridized with 1 μg each of both Cy3- and Cy5-labeled denatured cDNA and washed according to the Roche NimbleGen protocol. At least three, in most cases four, biological replicates were performed for each time point for each genotype. The arrays were scanned with a GenePix4000B microarray scanner system (Molecular Devices, Inc., Sunnyvale, CA, USA). The scanned images were initially quantified using Nimblescan (Roche) and the resulting data were normalized using the ArrayStar 3 (DNASTAR) software using the RMA quantile method.

### Data analysis

#### Proteome analysis

The spectral counts and unique peptide numbers from mass spectrometry were separately used to measure protein levels. Global normalization was performed using the trimmed mean method (that is, trimming the highest and lowest 5% of the values). Enrichment was assessed by comparing protein levels in PGCs (GFP-positive) and somatic cells (GFP-negative) at the 1-to-3 hour time point. To be identified as 'enriched' in 1-to-3 hour PGCs, a protein's levels had to satisfy the following criteria: 1) the two replicates of this protein in 1-to-3 hour PGCs both had a unique peptide number larger than or equal to two; 2) the geometric mean of the score (spectral count number or unique peptide number) for the two replicates of this protein in PGCs had to be larger than or equal to twice either the geometric mean of the replicates in the somatic cells or, if one of the somatic cell replicates had a zero score, the non-zero score. Those proteins whose levels were five-fold greater in PGCs versus soma (or completely absent in soma) were classified as 'PGC-specific'. Soma-enriched and specific proteins were defined following equivalent rules.

#### Transcriptome analysis

Analyses used the Significance Analysis of Microarrays (SAM) package. The genes significantly expressed in wild-type PGCs at 1-to-3 hours, 3-to-5 hours and 5-to-7 hours were separately determined by expression level at the corresponding time point (normalized to the mean expression level), using 'one class analysis' in SAM, with a false discovery rate (FDR) of 5% as the cutoff. The transcripts degraded or transcribed in PGCs were determined by comparing the expression level at two successive time points, using 'two class unpaired analysis' in SAM, with a FDR of 5% and fold change of two as the cutoff. The Smaug-dependent transcripts were determined by comparing the expression levels (normalized to the previous time point) in wild type and *smaug *mutants, using 'two class unpaired analysis', with a FDR of 5% and fold change of two as the cutoff. All the reported genes were restricted to the genes expressed significantly at the corresponding time points (see Additional file 27 for detailed information).

#### Gene Ontology term enrichment analysis

GO analysis was performed on the web server of the DAVID functional annotation tool [89]. A FDR of 10% was applied to evaluate the significance. The background setting for each analysis is shown in the relevant Additional file.

#### RBP binding-site enrichment

Two methods were applied to test for SRE enrichment in this study. In the first method [3], SREs were identified by searching for the CNGGN_(0-3) _consensus loop in a hairpin with a four base pair stem. The enrichment of transcripts containing at least one SRE was assessed using the hypergometric test. In the second method [67], enrichment for SREs was evaluated by comparing the accessibility of CNGG sites in the positive set versus negative set by measuring the area-under the receiver-operator characteristic (AUROC). If CNGG was absent from a transcript, an accessibility of zero was assigned. The significance level was assessed by calculating the Bonferroni-corrected Wilcoxon Mann Whitney *P*-value. Enrichment for Pumilio binding sites and AREs was assessed using the second method, with UGUAHAUA and AUUUA as the binding site, respectively.

#### microRNA target site enrichment

The targets of microRNA clusters and families were defined using PicTar and TargetScanFly, respectively [71,72]. Enrichment of miRNA target sites was assessed using the hypergeometric *P*-value, following multiple hypothesis testing. A FDR of 10% was applied to evaluate significance.

#### Network prediction

The network was predicted using the GeneMANIA web server [90], with the default settings using application version 3.0.7 and the 3 February 2012 version of the database.

### Data availability

The mass spectrometry data are available at ProteomeCommons.org Tranche network via the hash: 6ITDcEBOeJszDNlS5FLFu3pKPNiSSB8Q1tuCB2yBRTgSAFtx8JcVTnNFxvEwy6Wlz7gvQkIwUcXBisxbq6tKUHU1ne8AAAAAAAAWLQ = =. The microarray data are available at NCBI's Gene Expression Omnibus (GEO) website as series GSE34397.

## Abbreviations

ARE: AU-rich element; ARE-BP: ARE-binding protein; BDGP: Berkeley Drosophila Genome Project; CTD: carboxy-terminal domain of RNA polymerase II; FDR: false discovery rate; GFP: green fluorescent protein; GO term: Gene Ontology term; miR: microRNA; MuDPIT: multidimensional protein identification technology; MZT: maternal-to-zygotic transition; PBS: phosphate-buffered saline; PGC: primordial germ cell; RBP: RNA-binding protein; SAM: Significance Analysis of Microarrays; SRE: Smaug recognition element.

## Competing interests

The authors declare that they have no competing interests.

## Authors' contributions

NS, HL and HDL conceived and designed the project. NS and HL carried out all of the FACS purifications of PGCs. RNA purification and microarray experiments were conducted by NS and HL under the supervision of HDL and JTW. TK carried out the mass spectrometry on PGCs sorted by HL. XL carried out all of the data analysis under supervision of QM and HDL. AK carried out the RNA *in situ *hybridizations. AK and HH carried out the immunostains and confocal microscopy. HDL supervised the project and wrote the manuscript. All authors have read and approved the manuscript for publication.

## Supplementary Material

Additional file 1 **Distribution of the MuDPIT results**. An ordered plot showing the ratios of PGC:soma spectral counts for all of the proteins identified in this study (unique peptide number ≥2). The spectral counts shown in the plot are the geometric mean of the two replicates in the PGCs and somatic cells. The minimal measurement of all the spectral counts was added to each spectral count to avoid division by zero. The dashed lines indicate the thresholds used to determine PGC- or soma-specific (brown lines) and PGC- or soma-enriched proteins (black lines). PGC, primordial germ cell. Click here for file

Additional file 2 **Correlation between replicates**. **(a) **Spearman's correlation coefficients between mass spectrometry replicates. **(b) **Spearman's correlation coefficients between microarray replicates. Click here for file

Additional file 3 **All proteins detected by mass spectrometry**. The listed proteins had unique peptide number larger than 2, in either 1-to-3 hour GFP-positive or GFP-negative cells. GFP, green fluorescent protein. Click here for file

Additional file 4 **Supplementary text**. Click here for file

Additional file 5 **Supplementary methods**. Click here for file

Additional file 6 **PGC and somatic cell-enriched and -specific proteins**. **(a) **Proteins that are specific to (fold change ≥5) and enriched in (5 ≥ fold change ≥ 2) 1-to-3 hour somatic cells relative to 1-to-3 hour PGCs. **(b) **Proteins that are specific (fold change ≥5) and enriched (5 ≥ fold change ≥ 2) in 1-to-3 hour PGCs relative to 1-to-3 hour somatic cells. The lists were determined by comparing MuDPIT data for 1-to-3 hour GFP-positive and GFP-negative cells, using spectral counts as the measure of protein level. Asterisks indicate that the denominators of the fold change were zero. GFP, green fluorescent protein; MuDPIT, multidimensional protein identification technology; PGC, primordial germ cell. Click here for file

Additional file 7 **The somatic cell proteome**. A GeneMANIA-generated network seeded with the proteins specific to somatic cells at 1-to-3 hours and linked to the most relevant 20 proteins predicted by GeneMANIA. Soma-specific proteins according to our MuDPIT analysis are labeled in gray. In each case the 20 most relevant predicted proteins are labeled in white (if they were not detected by our MuDPIT analysis) or in orange (if they were detected by our MuDPIT analysis). The predictions of GeneMANIA were based on co-expression, co-localization, physical interaction and predicted interactions [90]. All the detected proteins had a unique peptide number larger than two in the results from mass spectrometry. MuDPIT, multidimensional protein identification technology. Click here for file

Additional file 8 **Gene Ontology (GO) term enrichment analysis of proteins enriched in PGCs or soma (listed in **Additional file 6**)**. **(a) **GO term results for the 1-to-3 hour PGC-specific proteins. **(b) **GO term results for the 1-to-3 hour PGC-enriched transcripts. **(c) **GO term results for the 1-to-3 hour soma-specific proteins. **(d) **GO term results for the 1-to-3 hour soma-enriched proteins. The proteins expressed in 1-to-3 hour somatic cells and PGCs served as the background sets. Terms with FDR < 10% were considered to be significant and are listed in the table. Click here for file

Additional file 9 **Transcripts expressed in PGCs or soma**. **(a) **Transcripts that are significantly expressed in PGCs at the 1-to-3 hour time point. The list was determined using the expression profiles of 1-to-3 hour PGCs. **(b) **Transcripts that are significantly expressed in PGCs at the 3-to-5 hour time point. The list was determined using the expression profiles of 3-to-5 hour PGCs. **(c) **Transcripts that are significantly expressed in PGCs at the 5-to-7 hour time point. The list was determined using the expression profiles of 5-to-7 hour PGCs. **(d) **Transcripts that are significantly expressed in the somatic cells at the 1-to-3 hour time point. The list was determined using the expression profiles of 1-to-3 hour GFP-negative cells. Click here for file

Additional file 10 **Transcripts enriched in PGCs or soma**. **(a) **Transcripts that are enriched in PGCs at the 1-to-3 hour time point. **(b) **Transcripts that are enriched in somatic cells at the 1-to-3 hour time point. The lists in (a, b) were determined by comparing the expression profiles of GFP-positive and GFP-negative cells at the 1-to-3 hour time point. All the reported transcripts have a FDR < 5% and the corresponding fold-change larger than two. Click here for file

Additional file 11 **Gene Ontology (GO) term enrichment analysis of transcripts enriched in PGCs or soma (listed in **Additional file 10**)**. **(a) **GO term results for the 1-to-3 hour PGC-enriched transcripts. The transcripts expressed in 1-to-3 hour PGCs served as the background set when performing the analysis. **(b) **GO term results for the 1-to-3 hour somatic-cell-enriched transcripts. The transcripts expressed in 1-to-3 hour somatic cells served as the background set. Terms with a FDR < 10% were considered to be the significant and are listed in the table. Click here for file

Additional file 12 **Comparison of the PGC proteome and transcriptome**. The log_2 _ratios of the geometric mean of the expression of PGC proteins (x-axis) and PGC mRNAs (y-axis) versus the soma. First, protein expression was normalized using the trimmed mean (see Materials and methods); second, the minimal expression level of the protein was added to avoid division by zero. Extra rules were applied to the lists shown in Additional files 6 and 10 (see Materials and methods). PGC, primordial germ cell. Click here for file

Additional file 13 **Heat maps showing the kinetics of the transcriptome during the maternal-to-zygotic transition in PGCs**. **(a) **Expression profile of PGC transcripts that decreased in level at the 3-to-5 hour time point relative to the 1-to-3 hour time point. **(b) **As in (a) but showing PGC transcripts that decreased in level at 5-to-7 hours relative to the 3-to-5 hour time point. **(c) **The expression profile of the PGC transcripts that increased in level at the 3-to-5 hour time point. **(d) **As in (c) but showing the PGC transcripts that increased at the 5-to-7 hour time point. PGC, primordial germ cell. Click here for file

Additional file 14 **Transcripts degraded during the PGC MZT**. **(a) **Transcripts that decreased in level at the 3-to-5 hour time point. The list was determined by comparing the expression profiles in PGCs at the 3-to-5 hour and 1-to-3 hour time points. **(b) **Transcripts that decreased in level at the 5-to-7 hour time point. The list was determined by comparing the expression profiles in PGCs at the 5-to-7 hour and 3-to-5 hour time points. All the reported transcripts have a FDR < 5% and the corresponding fold change larger than two. Click here for file

Additional file 15 **Transcripts in classes I to IX**. Detailed description of the nine classes is in the legend to Figure 4. The lists were obtained by overlapping the decay lists (Additional file 14), transcription lists (Additional file 19) and stable transcript lists (FDR > 30% and fold change < 1.2; Additional file 16) at the 3-to-5 hour and 5-to-7 hour time points. All transcripts probed by the array served as the background set. Terms with a FDR < 10% were considered to be the significant and are highlighted in yellow. Click here for file

Additional file 16 **Transcripts whose expression level remained constant in PGCs**. **(a) **Transcripts that are stable between the 1-to-3 and 3-to-5 hour time points. The list was determined by comparing the expression profiles in PGCs at 3-to-5 hour and 1-to-3 hour time points. **(b) **Transcripts that are stable between the 3-to-5 and 5-to-7 hour time points. The list was determined by comparing the expression profiles in PGCs at 5-to-7 hour and 3-to-5 hour time points. All the reported transcripts have a FDR > 30% and the corresponding fold change > 0.83 but < 1.2. Click here for file

Additional file 17 **GO term enrichment analysis of transcripts degraded during the PGC MZT (listed in **Additional file 14**)**. **(a) **GO term results for the transcripts that decrease in level at the 3-to-5 hour time point. The transcripts expressed in 1-to-3 hour PGCs served as the background set. **(b) **GO term results for the transcripts that decrease in level at the 5-to-7 hour time point. The transcripts expressed in 1-to-3 hour PGCs served as the background set. Terms with a FDR < 10% were considered to be the significant GO terms and are listed in the table. Click here for file

Additional file 18 **GO term enrichment analysis of transcripts in classes I to IX**. Transcripts are listed in Additional file 15. The transcripts expressed in 1-to-3 hour PGCs served as the background set for classes I, II, IV and V. All transcripts probed by the array served as the background set for classes III, VI, VII, VIII and IX. Click here for file

Additional file 19 **Transcripts that are zygotically transcribed in PGCs**. **(a) **Transcripts that increase in level at the 3-to-5 hour time point. The list was determined by comparing the expression profiles in PGCs at the 3-to-5 and 1-to-3 hour time points. **(b) **Transcripts that increase in level at the 5-to-7 hour time point. The list was determined by comparing the expression profiles in PGCs at the 5-to-7 and 3-to-5 hour time points. All the reported transcripts have a FDR < 5% and the corresponding fold change larger than two. Click here for file

Additional file 20 **GO term enrichment analysis of transcripts that are zygotically transcribed in PGCs (listed in **Additional file 19**)**. **(a) **GO term results for the transcripts that increased in level at the 3-to-5 hour time point. All transcripts probed by the array served as the background set. **(b) **GO term results for the transcripts that increased in level at the 5-to-7 hour time point. All transcripts probed by the array served as the background set. Terms with a FDR < 10% were considered to be the significant and are listed in the table. Click here for file

Additional file 21 **Transcripts that are degraded in PGCs in a Smaug-dependent manner**. **(a) **Transcripts that are Smaug dependent for decay in PGCs at 3-to-5 hour time point. The list was determined by comparing the expression profiles of the degraded transcripts (listed in Additional file 14) at the 3-to-5 hour time point (normalized to the expression level at the 1-to-3 hour time point), between wild-type and *smaug*-mutant PGCs. **(b) **Transcripts that are Smaug-dependent for decay in PGCs at the 5-to-7 hour time point. The list was determined by comparing the expression profiles of the degraded transcripts (listed in Additional file 14) at the 5-to-7 hour time point (normalized to the expression level at the 3-to-5 hour time point), between wild-type and *smaug*-mutant PGCs. All the reported transcripts have a FDR < 5% and the corresponding fold change larger than two. Click here for file

Additional file 22 **Smaug-dependent RNA decay in PGCs verified by *in situ *hybridization**. Four transcripts identified in the gene expression profiling experiment as Smaug-dependent for decay at 3-to-5 hours of embryogenesis were analyzed by fluorescence *in situ *hybridization in wild type and *smaug *mutants. **(a) ***spire*, **(b) ***orb*, **(c) ***mnt*, **(d) ***arrest*. Transcripts are shown in red and nuclei (DAPI) in blue. Left panels: 2-hour-old embryos. In all cases, transcripts are present at high levels in PGCs of wild type and *smaug *mutants. Right panels: 3-to-5 hour old embryos in which transcript levels have decreased in wild type but persist at high levels in *smaug *mutants. (Note: for *spire*, the wild-type embryo is less than 3 hours old, by which time transcripts have already disappeared.) Since *smaug *mutant embryos either do not undergo germ-band extension or undergo pseudo-extension, the PGCs remain at the posterior pole or move slightly dorso-anteriorly. DAPI, 4',6-diamidino-2-phenylindole; PGC, primordial germ cell. Click here for file

Additional file 23 **GO term enrichment analysis of transcripts that are degraded in PGCs in a Smaug-dependent manner (listed in **Additional file 21**)**. **(a) **GO term results for the transcripts that are Smaug-dependent for decay in PGCs at the 3-to-5 hour time point. The transcripts that are degraded in wild-type PGCs at the 3-to-5 hour were used as the background set. **(b) **GO term results for the transcripts that are Smaug-dependent for decay in PGCs at the 5-to-7 hour time point. The transcripts that are degraded in wild-type PGCs at 5-to-7 hour were used as the background set. **(c) **GO term results for the transcripts that are Smaug-dependent for decay in PGCs at the 3-to-5 hour time point. The transcripts that expressed in wild-type 1-to-3 hour PGCs were used as the background set. **(d) **GO term results for the transcripts that are Smaug-dependent for decay in PGCs at the 5-to-7 hour time point. The transcripts that were expressed in wild-type 1-to-3 hour PGCs were used as the background set. The terms with FDR < 10% were considered to be significant and are listed in the table. Click here for file

Additional file 24 **Transcripts that are transcribed in PGCs in a Smaug-dependent manner**. **(a) **Transcripts that are Smaug-dependent for transcription in PGCs at the 3-to-5 hour time point. The list was determined by comparing the expression profiles of the transcripts that increased in level (listed in Additional file 19) at the 3-to-5 hour time point (normalized to the expression level at the 1-to-3 hour time point), between wild-type and *smaug*-mutant PGCs. **(b) **Transcripts that are Smaug-dependent for transcription in PGCs at the 5-to-7 hour time point. The list was determined by comparing the expression profiles of the transcripts that increased in level (listed in Additional file 19) at the 5-to-7 hour time point (normalized to the expression level at the 3-to-5 hour time point), between wild-type and *smaug*-mutant PGCs. All the reported transcripts have a FDR < 5% and the corresponding fold change larger than two. Click here for file

Additional file 25 **GO term enrichment analysis of transcripts that are transcribed in PGCs in a Smaug-dependent manner (listed in **Additional file 24**)**. **(a) **GO term results for the transcripts that are Smaug-dependent for transcription in PGCs at the 3-to-5 hour time point. The transcripts that are transcribed at the 3-to-5 hour in wild-type PGCs were used as the background set. **(b) **GO term results for the transcripts that are Smaug-dependent for transcription in PGCs at the 5-to-7 hour time point. The transcripts that are transcribed at 5-to-7 hours in wild-type PGCs were used as the background set. **(c) **GO term results for the transcripts that are Smaug-dependent for transcription in PGCs at the 3-to-5 hour time point. All transcripts probed by the array were used as the background set. **(d) **GO term results for the transcripts that are Smaug-dependent for transcription in PGCs at the 5-to-7 hour time point. All transcripts probed by the array were used as the background set. GO terms with a FDR < 10% were considered to be significant and are listed in the table. Click here for file

Additional file 26 **microRNA target site enrichment in the different classes of PGC transcripts**. Targets of *miR *families and *miR *clusters were determined using Targetscan and PicTar, respectively. The listed *P*-values are uncorrected hypergeometric *P*-values, with a FDR < 10% after multiple test correction. Click here for file

Additional file 27 **Summary of the datasets derived from the microarray analyses**. This table summarizes all the datasets used in this study. Click here for file
