# Supplementary figures and images for: Genome-wide analysis of the maternal-to-zygotic transition in Drosophila primordial germ cells
Source: Genome Biol. 2012 Feb 20;13(2):R11. doi: 10.1186/gb-2012-13-2-r11 (PMC3334568; doi:10.1186/gb-2012-13-2-r11)

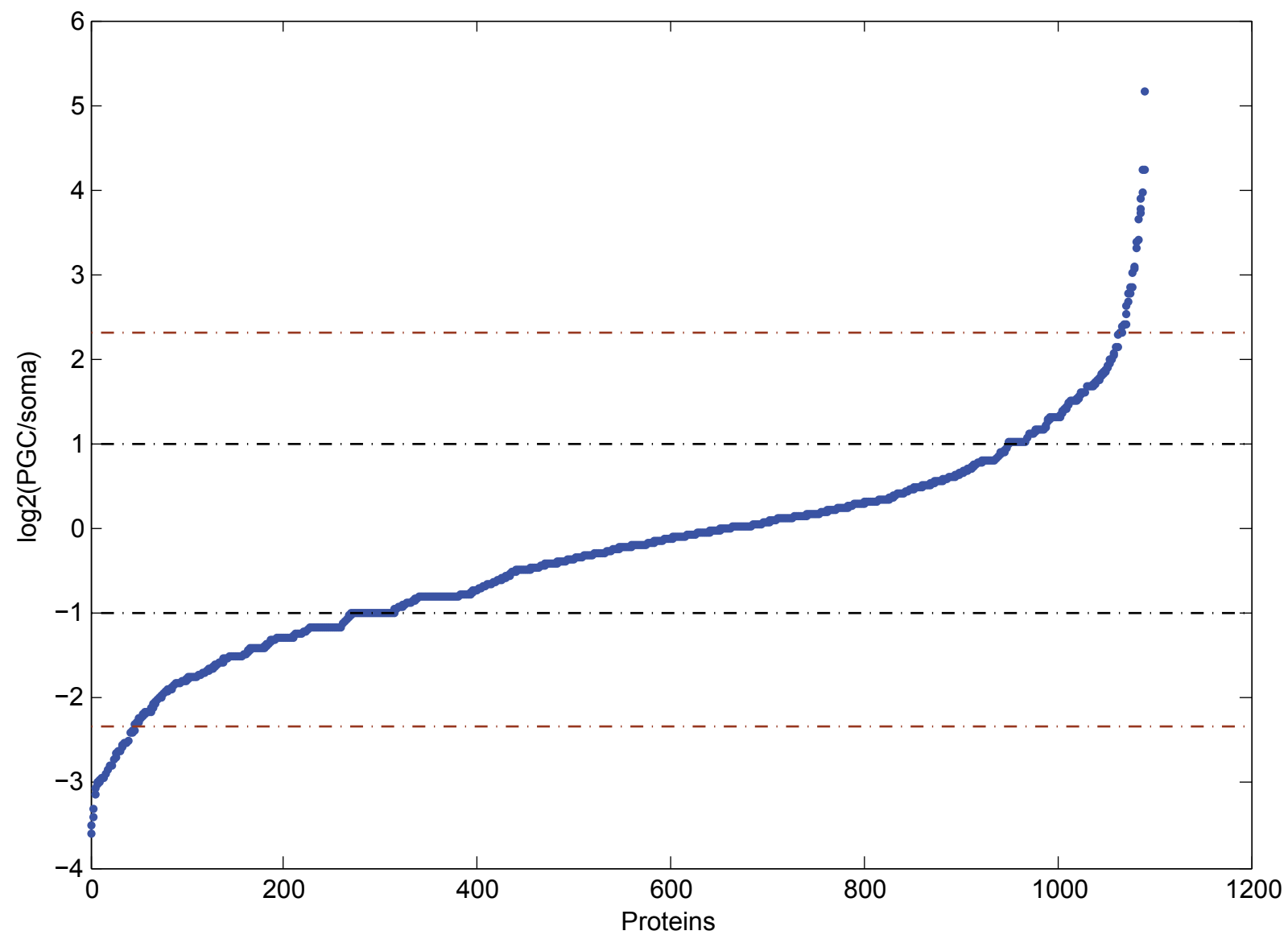

Supplement: Additional file 1 — Distribution of the MuDPIT results. An ordered plot showing the ratios of PGC:soma spectral counts for all of the proteins identified in this study (unique peptide number ≥2). The spectral counts shown in the plot are the geometric mean of the two replicates in the PGCs and somatic cells. The minimal measurement of all the spectral counts was added to each spectral count to avoid division by zero. The dashed lines indicate the thresholds used to determine PGC- or soma-specific (brown lines) and PGC- or soma-enriched proteins (black lines). PGC, primordial germ cell. [file gb-2012-13-2-r11-S1.PDF]

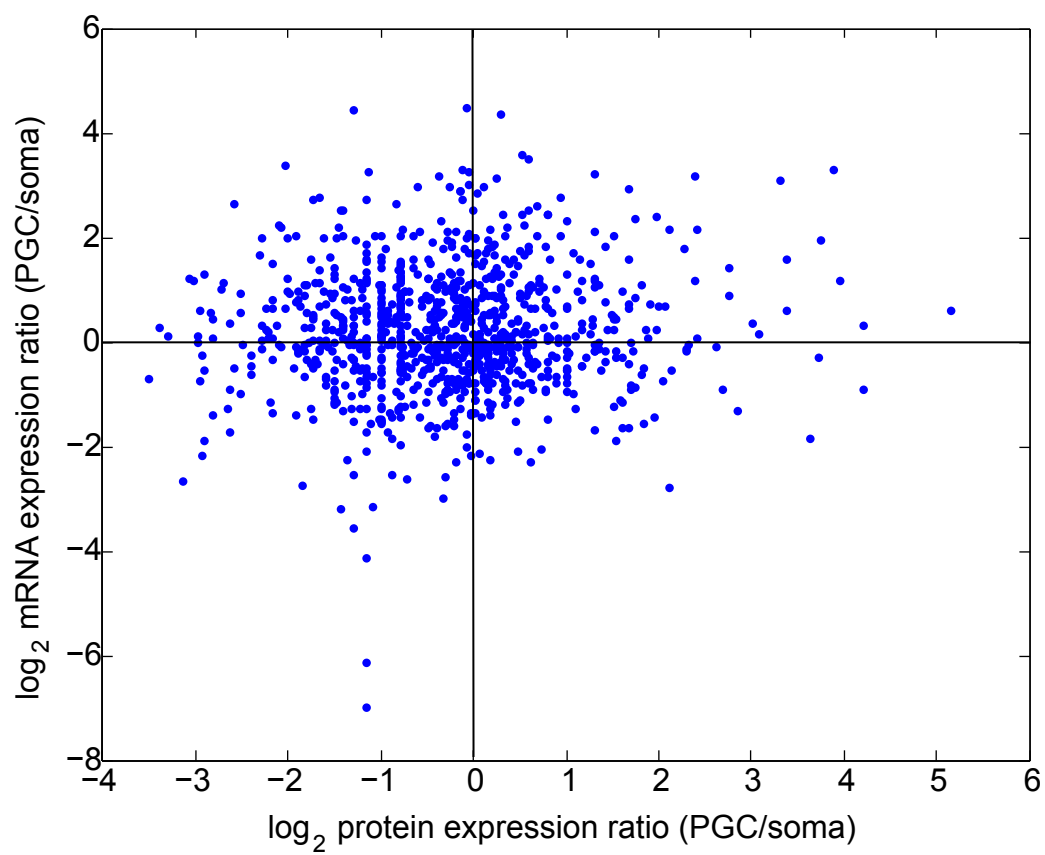

Supplement: Additional file 12 — Comparison of the PGC proteome and transcriptome. The log2 ratios of the geometric mean of the expression of PGC proteins (x-axis) and PGC mRNAs (y-axis) versus the soma. First, protein expression was normalized using the trimmed mean (see Materials and methods); second, the minimal expression level of the protein was added to avoid division by zero. Extra rules were applied to the lists shown in Additional files 6 and 10 (see Materials and methods). PGC, primordial germ cell. [file gb-2012-13-2-r11-S12.PDF]

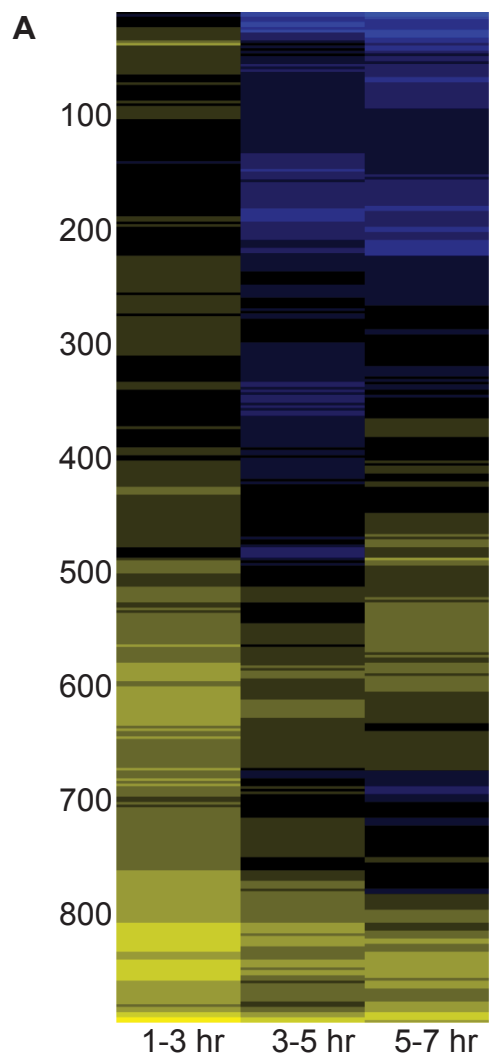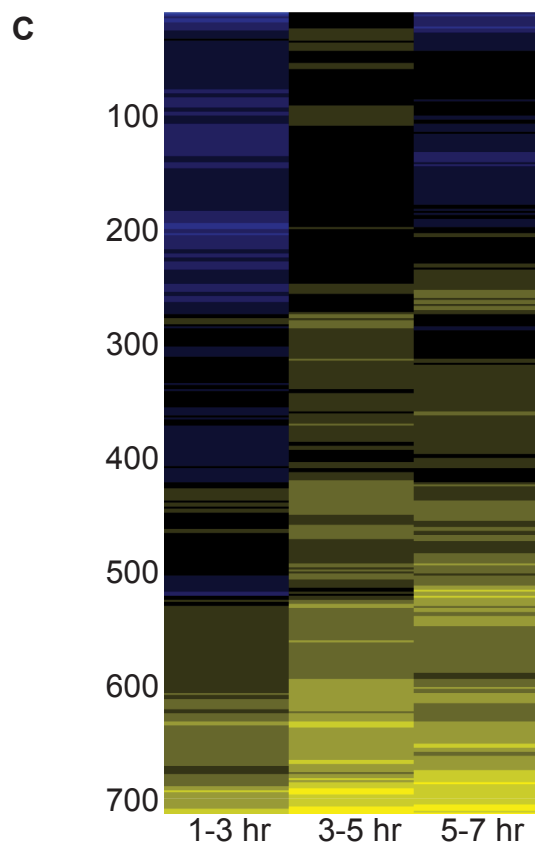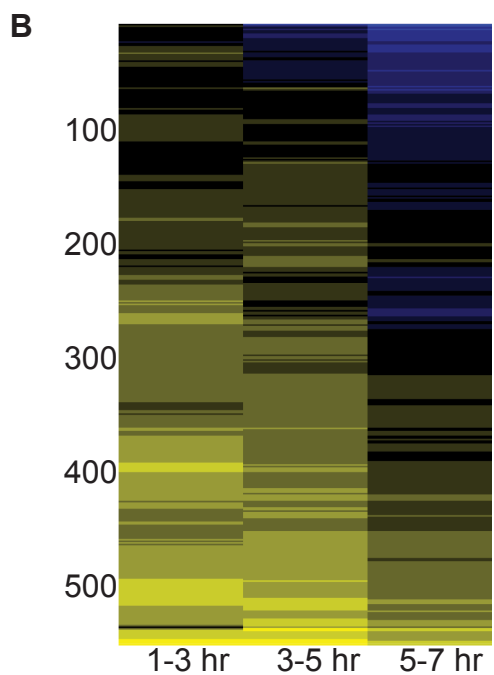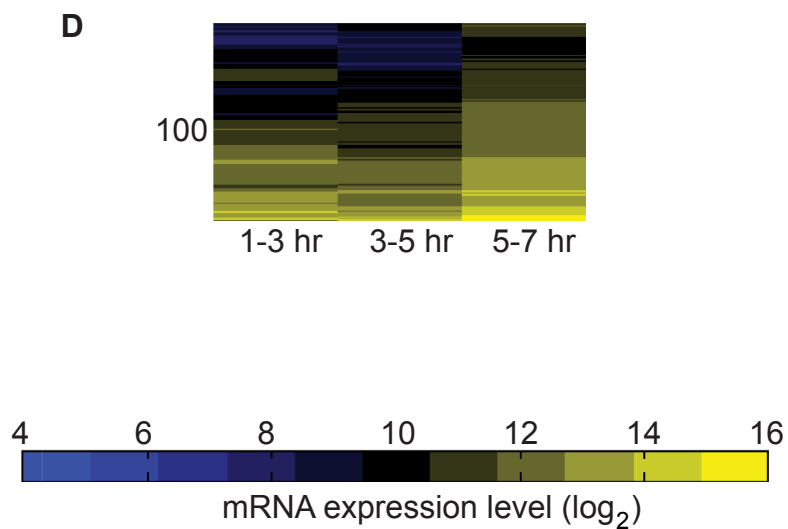

Supplement: Additional file 13 — Heat maps showing the kinetics of the transcriptome during the maternal-to-zygotic transition in PGCs. (a) Expression profile of PGC transcripts that decreased in level at the 3-to-5 hour time point relative to the 1-to-3 hour time point. (b) As in (a) but showing PGC transcripts that decreased in level at 5-to-7 hours relative to the 3-to-5 hour time point. (c) The expression profile of the PGC transcripts that increased in level at the 3-to-5 hour time point. (d) As in (c) but showing the PGC transcripts that increased at the 5-to-7 hour time point. PGC, primordial germ cell. [file gb-2012-13-2-r11-S13.PDF]

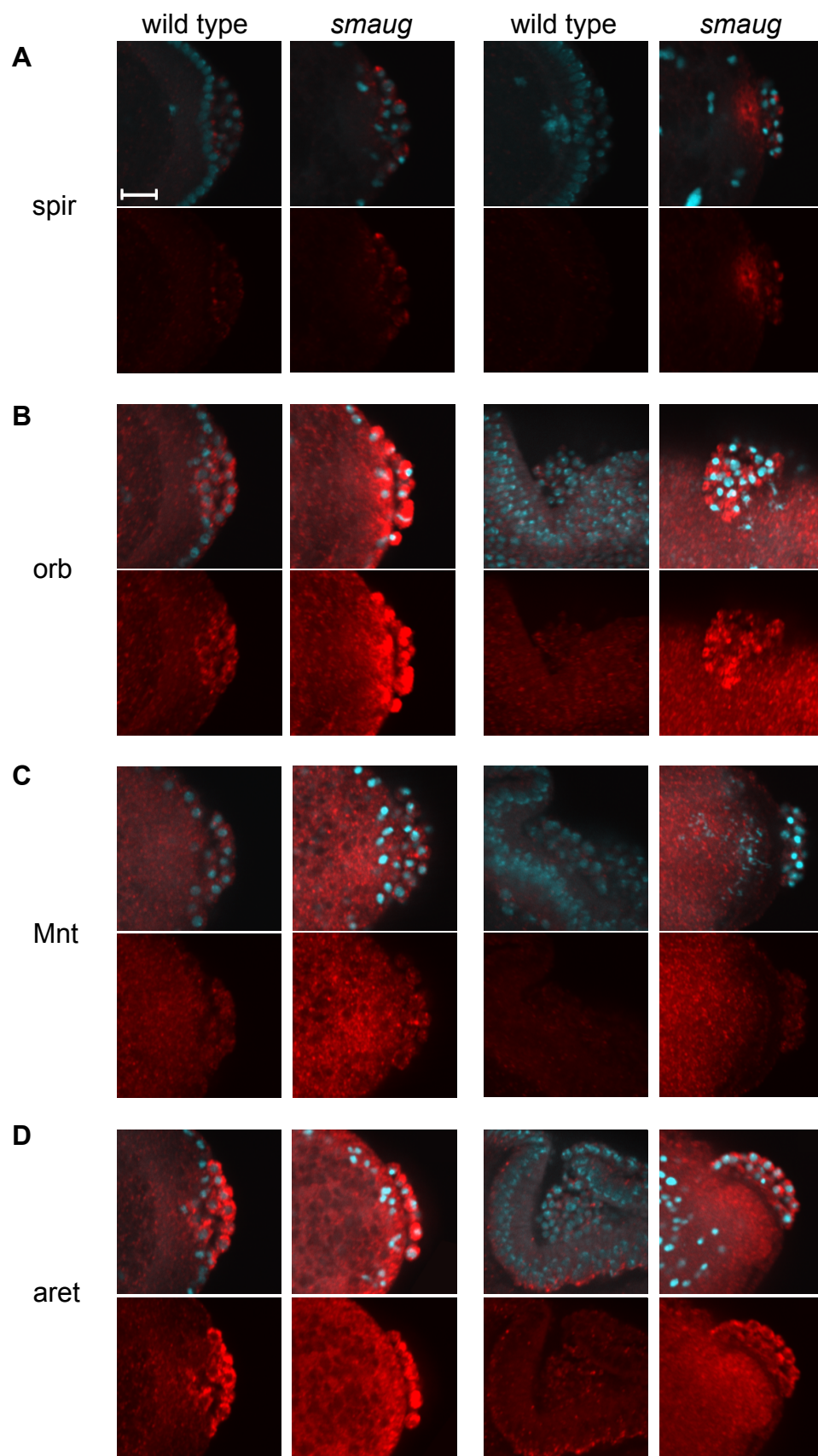

Supplement: Additional file 22 — Smaug-dependent RNA decay in PGCs verified by in situ hybridization. Four transcripts identified in the gene expression profiling experiment as Smaug-dependent for decay at 3-to-5 hours of embryogenesis were analyzed by fluorescence in situ hybridization in wild type and smaug mutants. (a) spire, (b) orb, (c) mnt, (d) arrest. Transcripts are shown in red and nuclei (DAPI) in blue. Left panels: 2-hour-old embryos. In all cases, transcripts are present at high levels in PGCs of wild type and smaug mutants. Right panels: 3-to-5 hour old embryos in which transcript levels have decreased in wild type but persist at high levels in smaug mutants. (Note: for spire, the wild-type embryo is less than 3 hours old, by which time transcripts have already disappeared.) Since smaug mutant embryos either do not undergo germ-band extension or undergo pseudo-extension, the PGCs remain at the posterior pole or move slightly dorso-anteriorly. DAPI, 4',6-diamidino-2-phenylindole; PGC, primordial germ cell. [file gb-2012-13-2-r11-S22.PDF]
