# Supplementary material for: Genome-wide analysis of the maternal-to-zygotic transition in Drosophila primordial germ cells
Source: Genome Biol. 2012 Feb 20;13(2):R11. doi: 10.1186/gb-2012-13-2-r11 (PMC3334568; doi:10.1186/gb-2012-13-2-r11)
Supplement: Additional file 4 — Supplementary text. [file gb-2012-13-2-r11-S4.PDF]

SUPPLEMENTAL TEXT

Section 1: The remaining 177 proteins may represent proteins that are present at high levels in whole 1.0-to-3.0 hour embryos (our sample) but not in 0-to-1.5 hour embryos (Gouw et al.'s sample) [27]; that is, proteins present at substantially higher levels at 1.5-to-3.0 than at 0-to-1.5 hours (although we cannot exclude the possibility that these derive from methodological or data analytical differences between the two studies).

The verified PGC-enriched/specific proteins are largely involved in the assembly of and posttranscrciptional control in, the germ plasm (Piwi, Oskar, Vasa, Aubergine) while the unverified PGC-enriched proteins include known or predicted RNA-binding proteins or translation factors (Bancal, Zn72D and eIF-5A); a protein phosphatase (Pp1-87B); components of the DNA replication complex (Mcm7 and Rfc3); proteasome subunits (Pros-beta3 and Pros-alpha7); a chromatin-regulator (Su(var)205); and numerous ribosomal proteins (Figure 3).

Section 2: We suspect that the somewhat higher true-positive rate seen for BDGP relative to Fly-FISH is a consequence of the fact that the BDGP methods enzymatically amplified the RNA *in situ* hybridization signal, emphasizing any difference between PGCs and soma; thus, while the Fly-FISH database provides subcellular resolution, the BDGP database may be most useful in identifying enrichment in particular cell types.

Among the most PGC-enriched transcripts (i.e., the top 250; see Additional File 10) are those that encode known components of the germ plasm (Polar granule component, Trailer hitch, Germ cell-less, Pumilio, Nanos, Me31B, Tudor-SN), proteins that regulate cell survival and migration (Zero population growth, Fear of intimacy), proteins that control germ line sex determination (Sans fille), chromatin components (Heterogeneous

nuclear RNP at 87D, Su(var)205, Histone demethylase, Mod(mdg4), Heterogeneous nuclear RNP at 98DE, Heterogeneous nuclear RNP at 27C, His2A, His2B), translation factors (eF1beta, eF1alpha, eF1gamma, eIF-5, eEF1delta, eRF1, eIF-4A, eIF-2Balpha, eIF-1A, Paip2) as well as other well-studied PGC-localized transcripts (CyclinB, Hsp83).

The top enriched GO terms for somatic-cell-enriched mRNAs were: 'developmental protein', 'cell fate commitment/determination', 'embryonic morphogenesis', 'transcription factor activity', 'extracellular region' and 'plasma membrane' (Additional File 11).

The top-enriched GO terms when the PGC-enriched transcripts were compared to all transcripts in PGCs were: 'metabolism', 'glycolysis', 'mitochondria', 'meiotic cell cycle' and 'pole plasm' (Additional File 11).

Section 3: Transcripts that were destabilized at 3-to-5 hours included those encoding well-known germ plasm components and/or posttranscriptional regulators (Cappuccino, Exuperantia, Spire, Swallow, Orb, Germ cell-less, Matrimony, Tra2, Pop2, Arrest/Bruno, BicC, Egalitarian, Smaug, Oskar), cell cycle regulators (CyclinA, CyclinB, String, CDK4, Twine, Orc4, CyclinD) and proteins that function in signal transduction, cell death and tumorigenesis (Stem cell tumor, Atg7, InR, Wunen, Ced6, Brca2, Sarah, Dredd, SpnF, Fs(1)N, Corp, Methuselah, Debcl). Transcripts destabilized at 5-to-7 hours included ones that encode proteins that function in germ plasm/posttranscriptional control (Faf, Zfh1, BicD, Spire, mRNA-cap, Orb, Exu, Arrest), cell death (Drak, Nc), chromatin regulation (Hmt4-20, Kdm4B, Mcm10), the cell cycle (Twine), and stem cell fate (Bam, SNCF, Ovo).

Transcripts that decreased at the 3-to-5 hour time point exhibited three patterns of expression (Figure 4, Additional File 15): they continued to decrease at the 5-to-7 hour time point (Class I: 99, including ones encoding germ plasm components and posttranscriptional regulators such as Orb, Gcl, and BicC); they decreased but then remained at constant levels at the 3-to-5 and the 5-to-7 hour time points (Class II: 149, including ones encoding Egalitarian, Stem cell tumor, and Brca2 homolog); or they increased in level at the 5-to-7 hour time point (Class III: 28). Class III presumably represents maternal mRNAs that are also transcribed zygotically although we cannot exclude the possibility that subsets of the Class-I and -II transcripts are also transcribed zygotically but that their steady-state level did not increase because of ongoing decay.

Transcripts that decreased only at the 5-to-7 hour time point fell into two classes: those whose levels were constant at the first two time points but then decreased at the third (Class IV: 45, including ones encoding Caudal, Mei-9, Zfh1 and Nullo); and those that increased in level between the first two time points but then decreased at 5-to-7 hours (Class VII: 36, including ones encoding Mediator complex subunit 1, FMR1, CDK5alpha, and Eph receptor). Class VII is likely to represent zygotically transcribed mRNAs that are destabilized at the third time point and thus exhibit transient expression in PGCs. A final category was transcripts present at constant levels at all three time-points (Class V: 162, including Brat, Orc6, Huntingtin, Bruce, Staufen, RnpS1, CyclinC, CyclinJ, Twin/CCR4, Shaggy, Skittles and Hts) (Additional File 15).

GO terms enriched in transcripts destabilized at 3-to-5 hour included ‘intrinsic/integral to membrane’, ‘EGF-like domain’, ‘Ras/Ras GTPase’, ‘N-linked glycosylation’, ‘pole plasm RNA localization’, ‘pole plasm assembly’, ‘embryonic axis

specification' and 'alternative splicing' (Additional File 17); for those destabilized at 5-to-7 hour, 'intrinsic/integral to membrane', 'dephosphorylation', 'lipid catabolism' and 'splicing variant' (Additional File 17).

Significantly enriched GO terms (Additional File 18) for Class I transcripts were 'intrinsic/integral to membrane' and 'N-linked glycoslyation'; for Class II were 'lipid metabolism', 'biotin/lipoyl attachment', 'FAD binding' and 'ATP-grasp fold'; for Class III were 'purine (ribo)nucleotide biosynthesis', 'nitrogen compound biosynthesis', 'ATPase activity coupled to transmembrane transport'. Classes IV and VII did not meet the 10% FDR cutoff: Class IV's best term was 'mitochondrial substrate carrier' at 14% FDR; and Class VII's were 'phosphoprotein' and 'phosphorylation' (14%). Likewise, there were no significantly enriched GO terms for Class V (the best FDR was >20%).

Section 4: Among the transcripts that increased at 3-to-5 hours were many encoding ribosomal proteins (both cytoplasmic and mitochondrial), signaling molecules (Plutonium, Basket, Slik), germ-plasm components and posttranscriptional regulators (Gustavus, POP2). Those that increased at 5-to-7 hours included mRNAs encoding the posttranscriptional regulators (Glorund, Penguin).

The transcripts that increased at 3-to-5 hours were enriched for GO terms related to 'structural constituent of ribosome', 'translation', 'structural constituent of mitochondrial ribosome', 'mitochondrial membrane part', 'mitochondrial electron transport', and 'transcription' (Additional File 20). At the 5-to-7 hour time point enrichment for the GO terms related to ribosomes, translation and transcription (but not mitochondria) were maintained while 'Casein kinase II' and 'mitotic spindle organization' became significant (Additional File 20).

The newly synthesized mRNAs that fell under the 'transcription' GO term included mRNAs encoding subunits of Pol II itself (18kD subunit, Rpb11), two TBP-associated factors (13 and 10), three subunits of the Mediator complex (1, 7, 10) and other factors that associate with Mediator and chromatin remodeling complexes (Gas41/MLLT1/ANC1). Two of the transcripts encode subunits of RNA Polymerase I (RPB10, RpI12), which transcribes rRNAs.

Zygotic transcripts could be subdivided into several categories (Figure 4): Classes III and VII have already been discussed above (28 and 36 mRNAs, respectively); Class IX transcript levels increased at both time-points (10 mRNAs); Class VIII transcript levels increased at 3-to-5 hours and then remained constant (82 mRNAs); Class VI transcript levels were constant between the first two time-points and then increased at 5-to-7 hours (18 mRNAs).

GO terms (Additional File 18) enriched in Classes III and VII have been discussed above. Class VI showed no significant GO terms (terms related to ribosomes were top, at 27% FDR). Class IX showed significant enrichment for 'Casein kinase II' and 'spermatogenesis/male gamete generation'; Class VIII for 'transcription from RNA polymerase II promoter', 'spliceosome', 'RNA biosynthetic process', 'large ribosomal subunit' and 'membrane enclosed organelle lumen'.

Section 5: Smaug-dependent transcripts at 3-to-5 hours included those encoding germ-plasm components and posttranscriptional regulators (Arrest/Bruno, Exuperantia, Orb, Spire, BicC, and Smaug itself) as well as molecules related to cell signaling, cell death and stem cell fate (Fs(1)Nasrat, Cappuccino, Ced6, Otu, Cdc42, Numb, Bbx, Stet, Mnt,

InR, CKIIbeta, CKIalpha). At 5-to-7 hour these included transcripts encoding Bam, SNCF, Hmt4-20, BicC, and Gcl.

GO term analysis (Additional File 23) indicated that transcripts dependent on Smaug for degradation at the 3-to-5 hour time-point, when compared to all RNAs in 1-to-3 hour PGCs, were enriched for ‘alternative splicing’, ‘pole-plasm RNA localization’, ‘stem cell division’, ‘integral/intrinsic membrane protein’, ‘EGF’, ‘sterile alpha motif SAM’, and ‘specific RNA polymerase II transcription factor activity’. When compared to all RNAs that are degraded between the first two time-points, enrichment was found for ‘alternative splicing’, ‘developmental protein’ and ‘cell surface receptor-linked signal transduction’. For Smaug-dependent transcripts at the 5-to-7 hour time-point (compared to all RNAs that are degraded at this time-point) only a single GO term was significant, ‘electron carrier activity’. GO term analyses on the individual subclasses of Smaug-dependent unstable transcripts did not yield any significant terms, most likely because of the small sizes of the groups of affected transcripts.

The Smaug-dependent ‘pole-plasm RNA localization’ GO-term transcripts included ones encoding Cappuccino, Cap-n-collar, Orb, Fs(1)K10, Spire, Exuperantia, and Rac2; the ‘stem cell division’ transcripts ones encoding Kokopelli – a CDK regulator implicated in stem cell division in the male germline, Numb – a regulator of Notch signaling, InR – the insulin receptor ortholog in flies, Cyclin A, Thickveins – a TGFbeta receptor, Jaguar – a cytoskeleton-associated protein, Snail – a Zinc-finger transcription factor, and Arrest/Bruno – an RBP known to regulate germ plasm transcripts such as *oskar*; the ‘specific RNA polymerase II transcription factor activity’ transcripts encoding Lola – a Zinc-finger/BTB-containing transcription factor recently implicated in gonad assembly in

the embryo; Trithorax-like – which encodes GAGA-factor and regulates chromatin, Tramtrack – a Zinc-finger/BTB transcriptional repressor that interacts with Trithorax-like and represses GAGA-mediated gene activation, Scalloped – a TEAD/TEF-family transcription factor that regulates genes involved in cell proliferation and growth, Erect wing – an NRF-related transcription factor, and Snail.
