# Supplementary material for: Genome-wide analysis of the maternal-to-zygotic transition in Drosophila primordial germ cells
Source: Genome Biol. 2012 Feb 20;13(2):R11. doi: 10.1186/gb-2012-13-2-r11 (PMC3334568; doi:10.1186/gb-2012-13-2-r11)
Supplement: Additional file 5 — Supplementary methods. [file gb-2012-13-2-r11-S5.PDF]

SUPPLEMENTAL METHODS

Fly stocks, maintenance and embryo collection:

All stocks were maintained at 25°C. To collect embryos for pole cell isolation, flies were grown in cages made from 500 ml plastic beakers fitted with a 10 cm petri-dish agar plate (25% apple juice, 0.15% methyl paraben, 0.625% sucrose and 2% agar) on the open side of the beaker. To prevent over-crowding of flies in the cage, each cage was seeded with a monolayer of anesthetized flies covering the bottom of the beaker. One- to two-day old flies of the desired genotype were kept in fresh bottles supplemented with dry yeast for 2-3 days before transferring to the cages. To improve egg lay efficiency, approximately a 70% : 30% female : male ratio was maintained by removing excess males. Fresh room temperature plates with wet yeast paste were used for all embryo collections. The first 2-hour embryo collection plate was discarded to maximize embryos synchronized in development during subsequent collections. Embryos were collected for 2 hours and then aged for 1, 3, or 5 hours at 25°C (for 1-to-3, 3-to-5 and 5-to-7 hour collections, respectively).

Fluorescence-activated cell sorting (FACS) of PGCs:

Cells were sorted using a BDFACS Aria (BD Biosciences) flow cytometer fitted with a 100 mm nozzle at 35 psi. Samples were sorted at 4°C and the sorted cells were collected into 1.5 ml of S2 medium at 4°C. Post-sort purity of GFP-positive cells was greater than 98% in the collected samples. The yield for VASA-GFP positive cells ranged from 25,000 to 110,000 cells/sort, starting from 8 cages of flies.

Mass spectrometry

Materials: Ultrapure grade formic acid was obtained from SIGMA, Canada. Ultrapure grade urea, ammonium carbonate, ammonium acetate, and calcium chloride, were from BioShop Canada Inc. HPLC grade solvents (methanol, acetonitrile and water) were obtained from Fisher Scientific, Canada. Recombinant, proteomics grade trypsin was from Promega, Madison, WI.

Sample preparation: The cells were incubated in hypotonic lysis buffer containing 10 mM HEPES, pH 7.4 for 30 minutes on ice. The suspension was briefly sonicated and 8 M urea, 100 mM Tris-HCl, pH 8.5 was added to reach a final urea concentration 1.5M, followed by incubation at 37C for 1 h. Calcium chloride was added to a final concentration of 2 mM. Proteins were digested with a 1:40 ratio of proteomics grade trypsin at 37°C overnight. Protein digestion was stopped by addition of 50 μL 2.5 % TFA. For peptide concentration and removal of salts, samples were solid phase extracted using Varian OMIX cartridges (Mississauga, ON, Canada) according to the manufacturers instructions and stored at -80C until further use.

Mass spectrometric analysis: A fully automated 5-cycle MuDPIT was performed as described (Taylor et al., 2009). Briefly, a nano-HPLC (Proxeon Biosystems, Odense, Denmark) was interfaced with a LTQ-Orbitrap XL mass spectrometer (Thermo Fisher Scientific, San Jose, CA), which is equipped with a nano-electrospray source (Proxeon Biosystems, Odense, Denmark). An analytical column was made by pulling a 75 μm i.d. fused silica microcapillary (Innova-Quartz, Phoenix, AZ) column to a fine tip (allowing for electrospray formation) with a P2000 laser puller (Sutter Instruments, Novato, CA). The column was then packed with ~10 cm of 5 μm Magic C18 100Ǻ reversed phase material (Michourom Bioresources Inc., Auburn, CA) with an in-house pressure vessel.

A Kasil fritted pre-column (150 µm i.d.) was packed with ~4 cm of 5 µm Magic C18 100Å reversed phase material (Michourom Bioresources Inc, Auburn, CA) followed by ~ 4 cm of Luna® 5µm SCX 100Å strong cation exchange resin (Phenomenex, Torrance, CA) by means of an in-house pressure vessel. This vented-column set-up (Licklider et al., 2002) was placed in-line with the EasyLC system and connected via a microsplitter tee (Proxeon Biosystems, Odense, Denmark) to which a distal voltage of 2.2 kV was applied. 15 µl of acidified sample was then automatically loaded from a 96-well microplate autosampler using the EASY-nLC system (Proxeon Biosystems, Odense, Denmark). The sample loading was followed by a 120 minutes HPLC gradient consisting of buffer A (water/0.1% formic acid) and buffer B (acetonitrile/0.1 % formic acid) at a flow of 400 nl/min, similar as recently reported (Taylor et al., 2009). After the sample loading step, consecutive "salt bumps" with 8 µl of 100 mM, 200 mM, 300 mM, and 500 mM NH_4Ac were loaded, followed by the same HPLC gradient as above. The MS functions were controlled by the XCalibur data system (Thermo Fisher Scientific, San Jose, CA) and the chouromatographic conditions by the Easy-LC software. Samples were analyzed on a LTQ-Orbitrap XL. The instrument method consisted of one MS full scan (400-1800 m/z) in the Orbitrap mass analyzer, an automatic gain control target (AGC target) of 500,000 with a maximum ion injection of 500 ms, 1 microscan and a resolution of 60,000 and using the preview scan option. Five data-dependent MS/MS scans were performed in the linear ion trap using the five most intense ions at 35 % normalized collision energy. The MS and MS/MS scans were obtained in parallel, with the ions selected for fragmentation (in the LTQ) from a preview scan and a more precise mass determination of a full MS scan (in the Orbitrap). AGC targets for the LTQ were 10,000 with a maximum ion

injection time of 100 ms. A minimum ion intensity of 1,000 was required to trigger a MS/MS spectrum. The dynamic exclusion was applied using a maximum exclusion list of 500 with one repeat count with a repeat duration of 30 seconds and exclusion duration of 45 seconds.

Database searches and protein identification: Raw data were converted to m/zXML using ReAdW and searched by Sequest Sorcerer™ (version 3.5) against a NCBI drosophila melanogaster protein sequence database (14331 sequences). The protein sequence database also contained common contaminants (human keratins and trypsin). The search was performed with a fragment ion mass tolerance of 0.4 Da, a parent ion mass tolerance of 50 ppm. Complete tryptic digest was assumed accepting a single missed cleavage site. Oxidation of methionine was specified as a variable modification. Validation of MS/MS-based peptide and protein identifications was performed using the Scaffold software (version Scaffold 2.1.1, Proteome Software Inc., Portland, OR). Peptide identifications were accepted if they could be established at greater than 95.0% probability as specified by the Peptide Prophet algorithm (Keller et al., 2002). Protein identifications were accepted if they could be established at greater than 99.0% probability and contained at least 1 identified peptides. Protein probabilities were assigned by the Protein Prophet algorithm (Keller et al., 2002). Proteins that contained similar peptides and could not be differentiated based on MS/MS analysis alone were grouped to satisfy the principles of parsimony.
