# Supplementary material for: Genome-wide analysis of the maternal-to-zygotic transition in Drosophila primordial germ cells
Source: Genome Biol. 2012 Feb 20;13(2):R11. doi: 10.1186/gb-2012-13-2-r11 (PMC3334568; doi:10.1186/gb-2012-13-2-r11)
Supplement: Additional file 7 — The somatic cell proteome. A GeneMANIA-generated network seeded with the proteins specific to somatic cells at 1-to-3 hours and linked to the most relevant 20 proteins predicted by GeneMANIA. Soma-specific proteins according to our MuDPIT analysis are labeled in gray. In each case the 20 most relevant predicted proteins are labeled in white (if they were not detected by our MuDPIT analysis) or in orange (if they were detected by our MuDPIT analysis). The predictions of GeneMANIA were based on co-expression, co-localization, physical interaction and predicted interactions [90]. All the detected proteins had a unique peptide number larger than two in the results from mass spectrometry. MuDPIT, multidimensional protein identification technology. [file gb-2012-13-2-r11-S7.PDF]

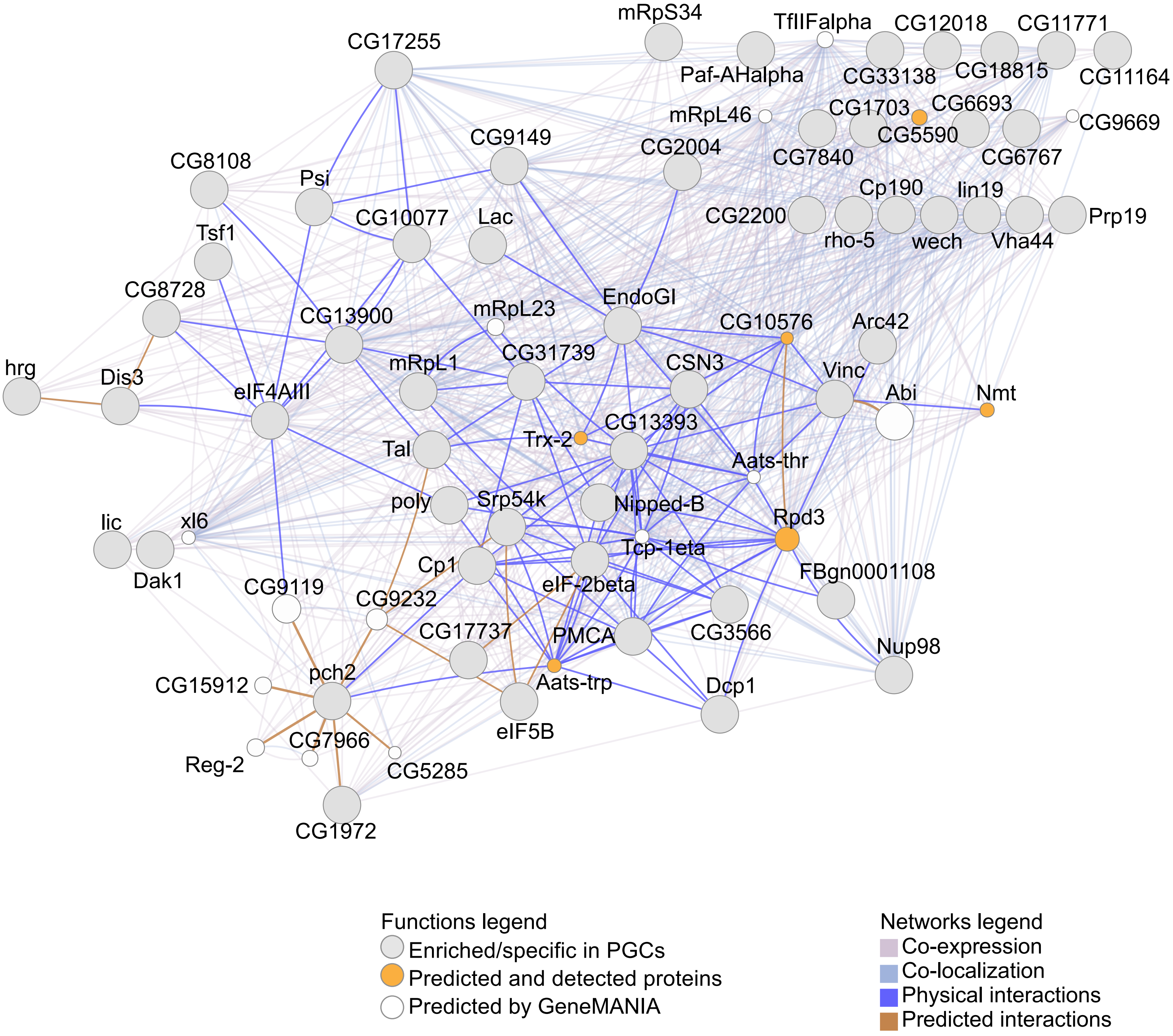

Additional File 7
